# AEP-cleaved DDX3X induces alternative RNA splicing events to mediate cancer cell adaptation in harsh microenvironments

**DOI:** 10.1172/JCI173299

**Published:** 2024-02-01

**Authors:** Wenrui Zhang, Lu Cao, Jian Yang, Shuai Zhang, Jianyi Zhao, Zhonggang Shi, Keman Liao, Haiwei Wang, Binghong Chen, Zhongrun Qian, Haoping Xu, Linshi Wu, Hua Liu, Hongxiang Wang, Chunhui Ma, Yongming Qiu, Jianwei Ge, Jiayi Chen, Yingying Lin

**Affiliations:** 1 Brain Injury Center, Shanghai Institute of Head Trauma and; 2 Shanghai Cancer Institute, Ren Ji Hospital, Shanghai Jiao Tong University School of Medicine, Shanghai, China.; 3 Department of Radiation Oncology, Ruijin Hospital, Shanghai Jiao Tong University School of Medicine, Shanghai, China.; 4 Department of Neurosurgery, Ren Ji Hospital, Shanghai Jiao Tong University School of Medicine, Shanghai, China.; 5 Department of Neurosurgery, Shanghai Changhai Hospital, Naval Medical University, Shanghai, China.; 6 Fujian Key Laboratory for Prenatal Diagnosis and Birth Defects, Fujian Maternity and Child Health Hospital, Affiliated Hospital of Fujian Medical University, Fuzhou, Fujian, China.; 7 Department of Neurosurgery, The First Affiliated Hospital of Fujian Medical University, Fuzhou, Fujian, China.; 8 Department of Neurosurgery, The First Affiliated Hospital of USTC, Division of Life Sciences and Medicine, University of Science and Technology of China, Anhui, China.; 9 Department of Biliary-Pancreatic Surgery and; 10 Department of General Surgery, Ren Ji Hospital, Shanghai Jiao Tong University School of Medicine, Shanghai, China.; 11 Department of Orthopedics, Shanghai General Hospital of Shanghai Jiao Tong University, Shanghai, China.

**Keywords:** Oncology, Proteases

## Abstract

Oxygen and nutrient deprivation are common features of solid tumors. Although abnormal alternative splicing (AS) has been found to be an important driving force in tumor pathogenesis and progression, the regulatory mechanisms of AS that underly the adaptation of cancer cells to harsh microenvironments remain unclear. Here, we found that hypoxia- and nutrient deprivation–induced asparagine endopeptidase (AEP) specifically cleaved DDX3X in a HIF1A-dependent manner. This cleavage yields truncated carboxyl-terminal DDX3X (tDDX3X-C), which translocates and aggregates in the nucleus. Unlike intact DDX3X, nuclear tDDX3X-C complexes with an array of splicing factors and induces AS events of many pre-mRNAs; for example, enhanced exon skipping (ES) in exon 2 of the classic tumor suppressor *PRDM2* leads to a frameshift mutation of *PRDM2*. Intriguingly, the isoform ARRB1-Δexon 13 binds to glycolytic enzymes and regulates glycolysis. By utilizing in vitro assays, glioblastoma organoids, and animal models, we revealed that AEP/tDDX3X-C promoted tumor malignancy via these isoforms. More importantly, high AEP/tDDX3X-C/*ARRB1-*Δ*exon 13* in cancerous tissues was tightly associated with poor patient prognosis. Overall, our discovery of the effect of AEP-cleaved DDX3X switching on alternative RNA splicing events identifies a mechanism in which cancer cells adapt to oxygen and nutrient shortages and provides potential diagnostic and/or therapeutic targets.

## Abstract 2

<p>Hypoxia and nutrient deprivation triggers AEP-specific cleavage of DDX3X, which yields truncated DDX3X that facilitates alternative splicing events and allows cancer cell adaptation.</p>

## Graphical abstract

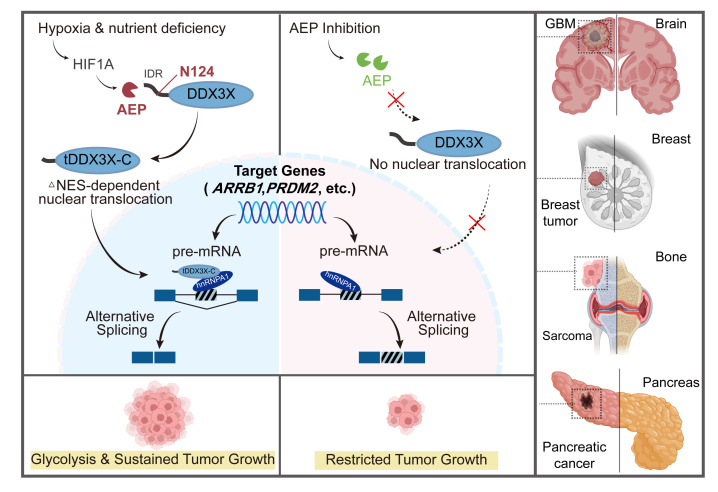

## Introduction

In the tumor microenvironment, cells often face a certain degree of oxygen and nutrient deprivation. Cancer cells strive to survive and proliferate in this harsh environment in multiple ways, including alternative splicing (AS) (1–3). Abnormal AS, which is mediated by complex splicing factors, is a key hallmark of tumors. Various RNA-Seq studies have shown that tumors are characterized by transcriptome-wide aberrant splicing and display approximately 30% more AS events than corresponding nonmalignant tumors, and these AS events allow cancer cells to create different protein isoforms that promote growth and survival (4). Although most recent studies have found that genetic mutations or epigenetic changes, as well as intratumoral transcriptional plasticity, contribute to malignant progression (5, 6), the signaling pathway regulating AS that is triggered by adverse tumor microenvironments remains elusive.

DEAD-box helicase 3 X-linked (DDX3X), one of the members of the DEAD (Asp-Glu-Ala-Asp)–box helicase family, participates in numerous aspects of eukaryotic RNA metabolism, including transcription, splicing, RNA export, and translation (7). Although DDX3X predominantly localizes to the cytoplasm, which is closely related to the efficiency of N-terminal leucine-rich nuclear export signal–dependent (NES-dependent) CRM1-mediated export (8), nuclear DDX3X has been found to predict poor outcomes in colorectal and breast cancers (9). Moreover, studies have revealed the role of DDX3X in tumorigenesis and tumor progression, but the precise function and underlying mechanisms are controversial (10–12).

Asparagine endopeptidase (AEP), also known as legumain (LGMN), is a cysteine proteinase belonging to the C13 family of peptidases (13). AEP is highly expressed and correlates with a poor prognosis and advanced clinical stage in various solid tumors, especially breast cancer and CNS tumors (14–16). Our previous work revealed that P53 and TMOD3 are important substrates of AEP in tumors (14, 17). However, while AEP is well studied in the context of neurodegenerative diseases as well as others (18–25), only a few pathological substrates for AEP have been discovered in tumors (26, 27). More substrates and the associated functions generated after AEP cleavage need to be systematically clarified.

In the present study, we discovered that hypoxia and nutrient deprivation triggered AEP-specific cleavage of DDX3X and identified the pathological functions and molecular mechanisms by which truncated DDX3X regulates AS to adapt to harsh microenvironments. Finally, the alternative isoforms *PRDM2-*Δ*exon 2* and *ARRB1-*Δ*exon 13* were identified, and the relationships among AEP/tDDX-3X-C*/ARRB1-*Δ*exon 13* in tumors and patient survival were further clarified.

## Results

### AEP interacts with and specifically cleaves DDX3X in response to hypoxia and nutrient deprivation in a HIF1A-dependent manner.

**Figure 1:**
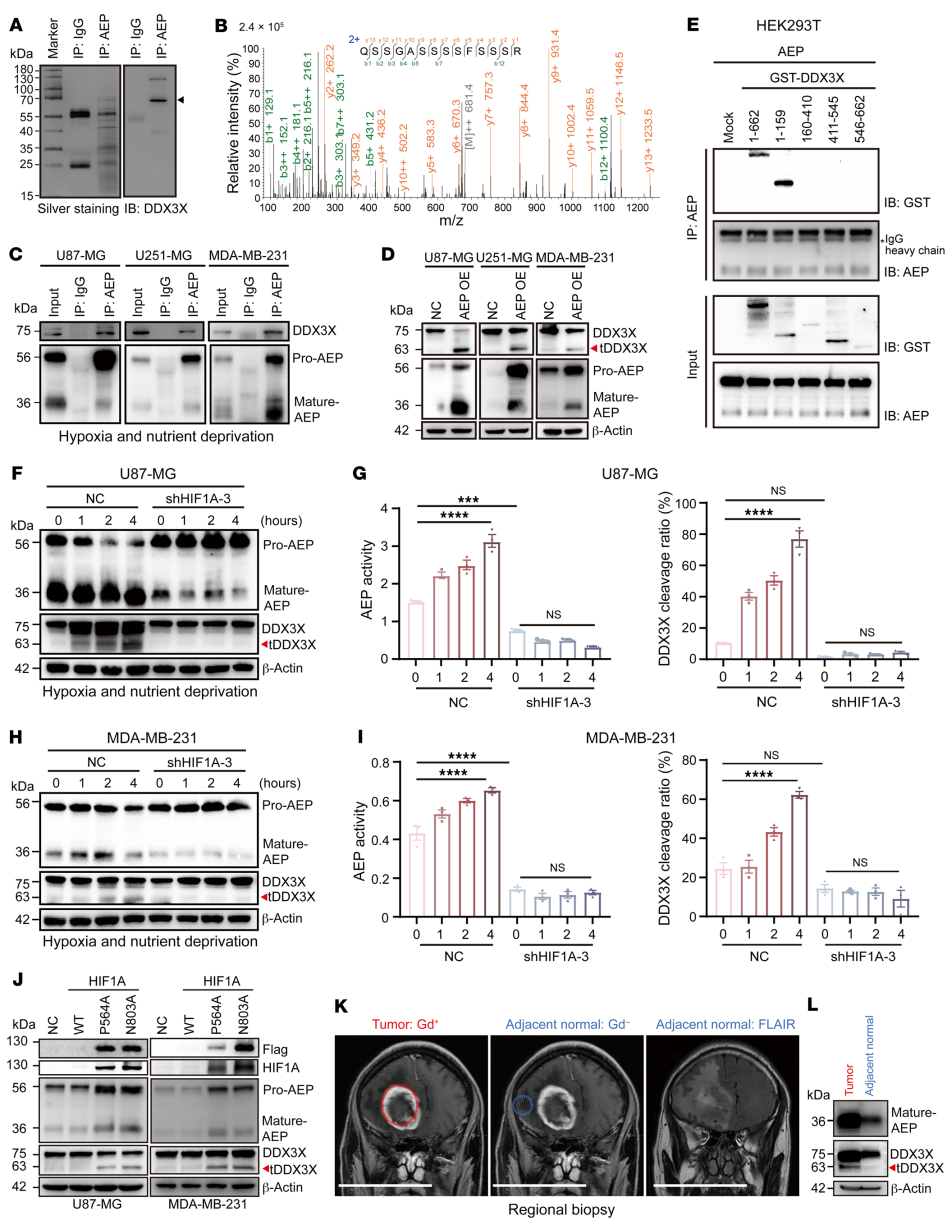
Hypoxia and starvation trigger AEP-specific cleavage of DDX3X in a HIF1A-dependent manner. (**A**) Left panel: silver-stained gel showing immunoprecipitated AEP or IgG and their bound proteins; right panel: immunoblot showing DDX3X in AEP immunoprecipitates. The arrowhead indicates that DDX3X may be a substrate of AEP. (**B**) MS analysis of proteins interacting with AEP. The detected MS/MS peptide spectrum of DDX3X is shown. (**C**) Endogenous co-IP assays detected the AEP/DDX3X interaction in U87-MG, U251-MG, and MDA-MB-231 cells underwent hypoxia and nutrient deprivation. (**D**) Immunoblots of DDX3X, AEP and β-actin in U87-MG, U251-MG and MDA-MB-231 cells with or without AEP overexpression (OE). Blots run contemporaneously are presented together. (**E**) Co-IP assays detected the ability of different DDX3X domains to interact with AEP. (**F**) Immunoblots of DDX3X, AEP and β-actin in hypoxia-and-starvation–stressed cancer cells (U87-MG) with or without KD of HIF1A. (**G**) Quantitation of the DDX3X cleavage ratio (tDDX3X/DDX3X) and AEP activity in the indicated U87-MG cells. (**H**) Immunoblots of DDX3X, AEP and β-actin in hypoxia-and-starvation–stressed cancer cells (MDA-MB-231) with or without KD of HIF1A. Blots run contemporaneously are presented together. (**I**) Quantitation of the DDX3X cleavage ratio (tDDX3X/DDX3X) and AEP activity in the indicated MDA-MB-231 cells. (**J**) Immunoblots of Flag, HIF1A, DDX3X, AEP and β-actin in U8 7-MG and MDA-MB-231 cells with WT or stable HIF1A mutant OE. (**K**) Representative Gd-enhanced T1-weighted and FLAIR MRI images of regional biopsy derived from tumor and adjacent normal parts of GBM. Scale bar: 10 cm. (**L**) Immunoblots of DDX3X, AEP, and β-actin of tumor and adjacent normal tissues of GBM. Data were plotted as the mean ± SEM. Statistical analysis was performed using 1-way ANOVA followed by Šidák’s multiple comparisons test (**G** and **I**). ***P < 0.001, ****P < 0.0001.

To identify important substrates of AEP, we conducted immunoprecipitation-mass spectrometry (IP-MS) with an anti-AEP antibody using *AEP*-KO mouse embryonic fibroblasts (MEFs) after the reexpression of AEP. IP-MS revealed that DDX3X might be a substrate of AEP (Figure 1, A and B). Although nuclear DDX3X has been found to predict poor outcomes in breast and colorectal cancer, the pathological functions of DDX3X in cancer are controversial (9, 12). We searched the Gene Expression Profiling Interactive Analysis (GEPIA) database. DDX3X was found to be significantly upregulated in glioma and pancreatic adenocarcinoma compared with the corresponding normal tissues (Supplemental Figure 1A; supplemental material available online with this article; https://doi.org/10.1172/JCI173299DS1). High DDX3X expression was associated with poor patient prognosis in breast cancer and glioma (Supplemental Figure 1B). Moreover, AEP and DDX3X had a significant positive correlation in breast cancer and glioma (Supplemental Figure 1C). We further measured the expression of DDX3X in different cell subtypes within the tumor by performing single-nuclear RNA-Seq (snRNA-Seq) for 6 cases of glioblastoma (GBM, the most malignant glioma, WHO IV grade). The results indicated that DDX3X was highly and ubiquitously expressed in most clusters (Supplemental Figure 1D).

We then verified the interaction of AEP and DDX3X by endogenous co-IP, especially in glioma (U87-MG and U251-MG) and breast cancer cells (MDA-MB-231) subjected to hypoxia and nutrient deprivation (Figure 1C). Moreover, elevated DDX3X cleavage was found in AEP-overexpressing cells in an array of solid tumor cells (Figure 1D and Supplemental Figure 1E). Furthermore, AEP with a mutation that eliminates its enzymatic activity (AEP C189S) failed to cleave DDX3X (Supplemental Figure 1F). Given that specific mutations in a protein may affect its biological function, we searched the Cancer Cell Line Encyclopedia (28) and collected 6 natural missense mutations of DDX3X in cancer (Supplemental Figure 1G). The corresponding mutant DDX3X was cotransfected with AEP into HEK293 cells. The P386S and L540M mutations substantially increased the cleavage of DDX3X; however, the T275M and S290L mutations led to substantial decreases in the DDX3X protein levels (Supplemental Figure 1H). Moreover, a domain assay revealed that the N-terminus of DDX3X (amino acids 1–159) is the major motif responsible for AEP binding (Figure 1E).

Since AEP is a stress response protease, we investigated the upstream trigger of AEP cleavage of DDX3X. As shown in Figure 1, F–I, hypoxia and starvation stimuli significantly enhanced the activation of AEP and tDDX3X levels in glioma and breast cancer cells, especially after 4 hours, which did not occur under a single stimulus, such as nutrient deprivation or hypoxia (Supplemental Figure 2A). Consistently, it was found that AEP specifically cleaved DDX3X in response to hypoxia and nutrient deprivation in pancreatic cancer cells (PANC-1) and osteosarcoma cells (143B) (Supplemental Figure 2, B and C). Moreover, we examined AEP and the corresponding DDX3X cleavage rates in multiple subtypes of breast cancer cell lines under conditions of hypoxia and nutrient deprivation. As shown in Supplemental Figure 2, D and E, in triple-negative (TN) breast cancer cells, there is a correlation between AEP activation and DDX3X cleavage, whereas in luminal A (LA), luminal B (LB), and HER-2 positive breast cancer cells, this correlation was somewhat reduced, but AEP activation and DDX3X cleavage bands were still observed under conditions of hypoxia and nutrient deprivation.

HIF-1α (HIF1A), a key factor stimulated by hypoxia, has been revealed as an important upstream regulator of AEP, and we investigated whether HIF1A mediates AEP cleavage of DDX3X. We analyzed the importance of HIF1A in glioma via TCGA and our tissue microarray. Both sets of data revealed that HIF1A was significantly upregulated in high-grade glioma (HGG, including WHO grade III and grade IV, referred to as GBM) compared with low-grade glioma (LGG, including WHO grade I and II) (Supplemental Figure 3, A and B). High HIF1A is associated with a poor prognosis for glioma patients (Supplemental Figure 3, C and D and Supplemental Tables 1 and 2). Moreover, after efficient knockdown (KD) of HIF1A (Supplemental Figure 3, E and F), AEP activation and tDDX3X were significantly suppressed (Figure 1, F–I), indicating that hypoxia and starvation promoted AEP-specific cleavage of DDX3X in a HIF1A-dependent manner. We further constructed mutant plasmids for HIF1A (HIF1A-P564A and HIF1A-N803A) to maintain HIF1A stability under normoxia (29, 30). As shown in Figure 1J, stable HIF1A substantially promoted AEP activation and DDX3X cleavage.

To further identify this phenomenon, we performed an MRI-guided localized biopsy of GBM tissue from a newly diagnosed IDH1-WT GBM patient. As shown in Figure 1K, the gadolinium (Gd)-enhancing lesion of the GBM represents the tumor area, and the T2-FLAIR image highlights the adjacent area. The tumor and adjacent normal tissue samples exhibited distinct histopathological characteristics (Supplemental Figure 3G). Western blot results demonstrated that mature AEP and cleaved DDX3X were found mainly in tumor compared to adjacent normal tissue (Figure 1L). Thus, these data indicate that AEP interacts with and specifically cleaves DDX3X in multiple kinds of tumor cells in response to HIF1A-mediated hypoxia and starvation stimuli.

### DDX3X is cleaved by AEP at Asn124, producing a relatively stable carboxyl-terminal DDX3X fragment.

**Figure 2:**
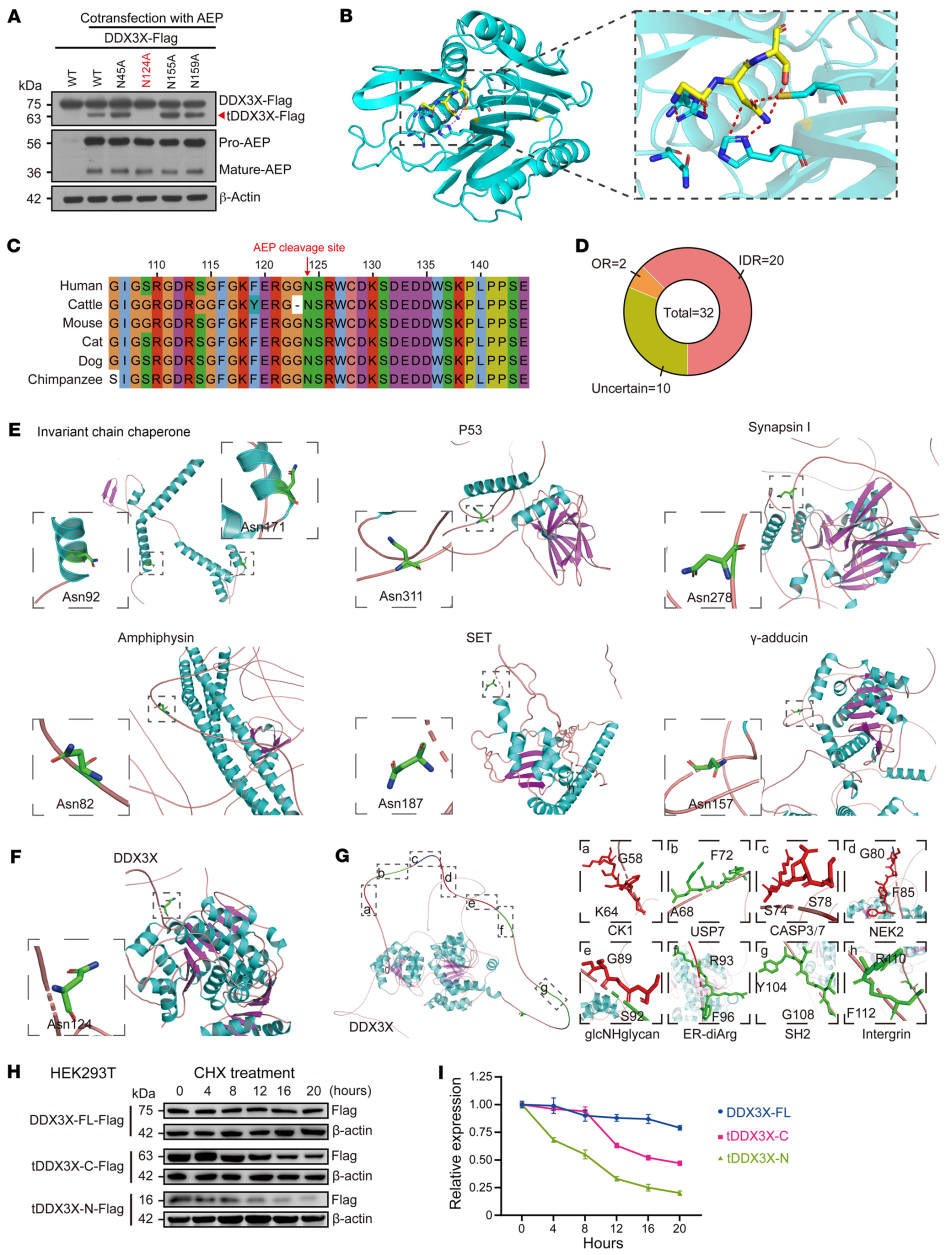
DDX3X was cleaved at the Asn124 site, which is located in the IDR, producing a relatively stable carboxyl-terminal fragment of DDX3X. (**A**) Immunoblots of Flag-tagged DDX3X, AEP, and β-actin in HEK293T cells cotransfected with AEP and DDX3X-WT or different point mutants of Asn (DDX3X-N45A/N124A/N155A/N159A). (**B**) Structure diagram of human AEP binding with DDX3X and the appropriate domains of DDX3X interacting with the AEP enzymatic center (human AEP pdb code: 4D3Z). (**C**) Sequence alignment of DDX3X amino acids among different species. (**D**) Percentage of AEP cleavage site–located regions, divided into the disordered region (IDR), ordered region (OR) and uncertain region. The regional discrimination of regional structure is based on literature retrieval combined with AlphaFold and Pondr-VSL2 prediction. (**E**) Cleavage-site view of the invariant chain chaperone, P53, synapsin I, amphiphysin, SET, and γ-adducin (sequence numbering following UniProt entries P04233, P04637, P17600, P49418, Q9EQU5, and Q9QYB5). (**F**) Cleavage-site view of DDX3X (sequence numbering following UniProt entries O00571). The IDR region is depicted in salmon. The side chains of Asn are shown as stick models. All panels were created within PyMOL with carbon depicted in green, oxygen in red, and nitrogen in blue. (**G**) The potential regulatory motif in the IDR region of DDX3X analysis by ELM. The red motifs included the potential modification site by the corresponding enzyme, and the green motifs included the potential combination site with the corresponding proteins. (**H**) A protein fragment stability assay was used to analyze the halflife of DDX3X and its cleavage fragment. Immunoblots of Flag-tagged DDX3X-FL, tDDX3X-N, tDDX3X-C and β-actin in HEK293T cells treated with 20 μg/mL cycloheximide (CHX) for the indicated time courses. (**I**) Degradation curves of DDX3X-FL, tDDX3X-N, and tDDX3X-C. Data were plotted as the mean ± SEM.

Due to the strict cleavage of asparagine by AEP, a panel of DDX3X mutants with asparagine (N) to alanine (A) substitution mutations were generated, and only the cells cotransfected with the DDX3X-N124A mutant and AEP failed to produce cleaved DDX3X (Figure 2A), confirming that AEP specifically cleaves DDX3X at Asn124. Through multispecies sequence alignment, N124 was found to be species conserved and precisely fit into the AEP enzymatic center (Figure 2, B and C).

In recent years, researchers have found important pathological functions of AEP and its associated substrates. For example, in Alzheimer’s disease (AD), activated AEP cleaves endogenous tau protein in the ageing brain, allowing tau fragments to form insoluble fibrils that are neurotoxic and lead to symptoms of AD (19). Additionally, AEP cleaves synapsin in the brains of patients with AD and generates the C-terminal synapsin I fragment, which is abnormally distributed in neurons and induces synaptic dysfunction (31). The pathologic importance of the synergistic regulation of AEP with its cleavage substrates is not unique; it has been reported in other neurodegenerative diseases (32), carcinomas [e.g., GBM (17)], thoracic aortic dissection (23), and other disorders. However, the regular pattern of AEP versatile substrates has never been studied. We collected all known substrates and associated cleavage sites (Supplemental Table 3). As shown by AlphaFold and Pondr-VSL2 (33, 34), we surprisingly found that AEP cleavage sites were located mainly in the intrinsic disordered region (IDR) (Table 1 and Figure 2D). In contrast, to cleave at the structural region in the invariant chain chaperone, representative cleavage in the IDR region of p53, synapsin I, amphiphysin, SET, and γ-adducin is shown (Figure 2E). Consistently, N124 of DDX3X was also located in the IDR (Figure 2F). To explore the regulatory functions of IDR in DDX3X cleavage, we used ELM, a computational biology resource for investigating candidate functional sites in eukaryotic proteins (35). ELM analysis revealed several potential regulatory motifs with different modification potentials in this region (Figure 2G).

Cleavage of DDX3X by AEP at N124 produces 2 protein truncations of DDX3X (DDX3X-1-124, referred to as tDDX3X-N, and DDX3X-125-662, referred to as tDDX3X-C). Since protease cleavage is associated with protein degradation, the protein stabilities of these 2 truncations were evaluated by a cycloheximide (CHX) chase protein stability assay. The results showed that full-length DDX3X (DDX3X-FL) had an intracellular half-life of more than 24 hours, whereas the stability of both truncations was inferior to that of DDX3X-FL. The half-life of tDDX3X-N was only 4 hours, while the half-life of tDDX3X-C was more than 16 hours, and nearly half of tDDX3X-C was still expressed in the cells after 24 hours (Figure 2, H and I), indicating that AEP-specific cleavage of DDX3X at N124 does not simply lead to degradation but may result in unexpected functions of tDDX3X-C in cancer. Thus, these data indicate that AEP specifically cleaves DDX3X at N124, which is located in the intrinsically disordered region and produces a relatively stable tDDX3X-C fragment.

### tDDX3X-C aggregates in the nucleus due to the absence of the N-terminal NES, and nuclear DDX3X is positively associated with AEP in HGG.

**Figure 3:**
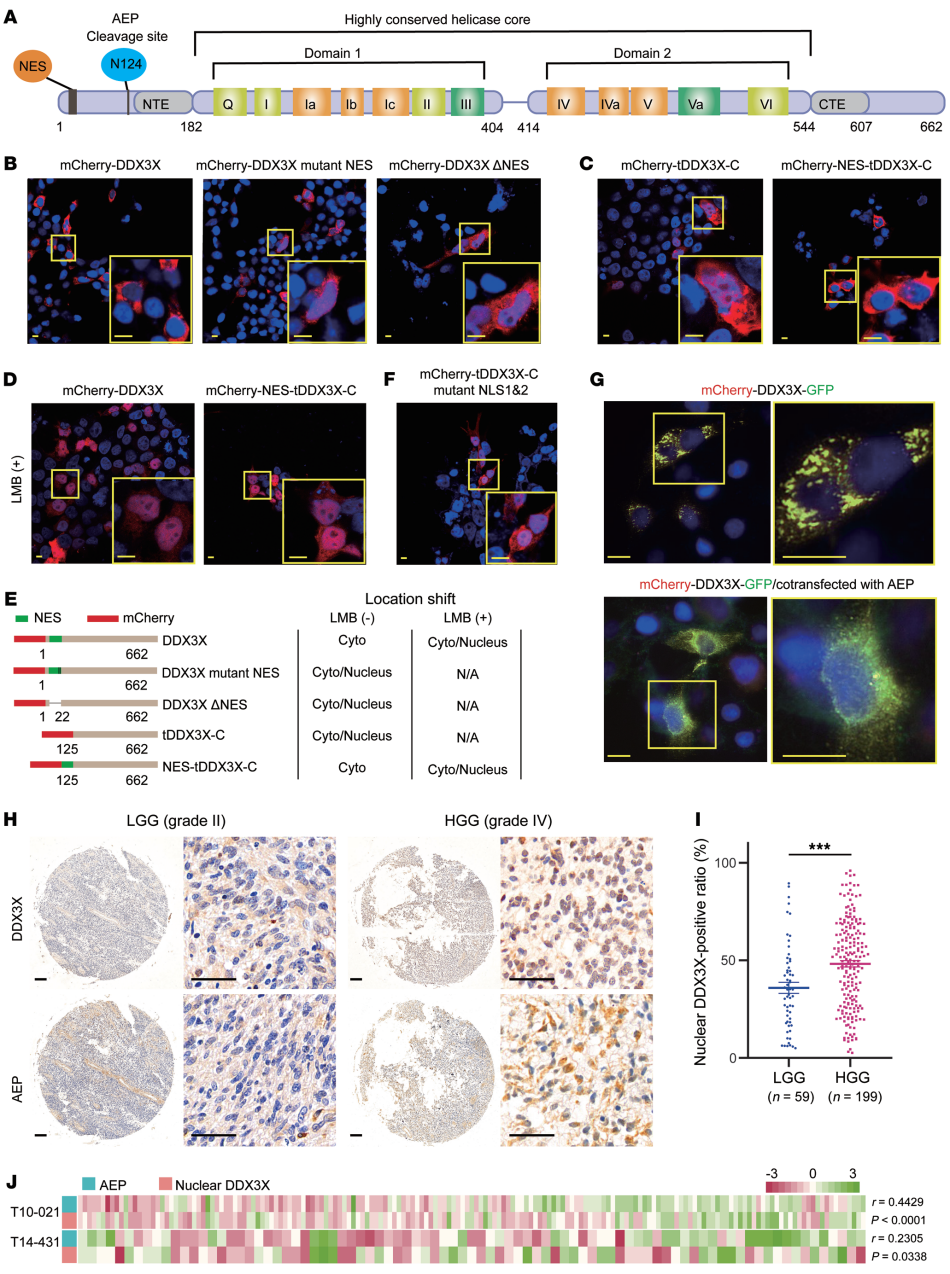
tDDX3X-C aggregates in the nucleus due to the absence of the N-terminal NES, and nuclear DDX3X is positively associated with AEP in HGG. (**A**) 2-dimensional structure diagram of DDX3X. (**B** and **C**) HEK293T cells were transfected with mCherry-DDX3X, mCherry-DDX3X mutant NES, mCherry-DDX3X ΔNES, mCherry-tDDX3X-C, and mCherry-NES-tDDX3X-C for 24 hours, fixed, counterstained with DAPI, and finally visualized by confocal microscopy. Representative images are shown. Scale bar: 10 μm. (**D**) HEK293T cells were transfected with mCherry-DDX3X and mCherry-NES-tDDX3X-C for 24 hours and then treated with LMB, an inhibitor of protein nuclear transport. The follow-up procedure was the same as before. Representative images are shown. Scale bar: 10 μm. (**E**) Schematic diagram of DDX3X and its series of mutants; the corresponding location shift is shown. (**F**) HEK293T cells transfected with mCherry-tDDX3X-C mutant NLS1 and 2. The follow-up procedure was the same as before. Representative images are shown. Scale bar: 10 μm. (**G**) HeLa cells transfected with mCherry-DDX3X-GFP were cotransfected with or without AEP for 24 hours, counterstained with DAPI and visualized by confocal microscopy. Representative images are shown. Scale bar: 10 μm. All images were processed by Leica Imaging System software. (**H**) Representative images of IHC staining of AEP and DDX3X in glioma tissue microarrays containing different WHO grades. Scale bars: 200 μm for overview; 50 μm for enlargement. (**I**) Scatter plots of the nuclear DDX3X-positive ratio in HGG and LGG. Quantitative analysis (H-index) of IHC results was performed by Aipathwell (Servicebio, Wuhan). (**J**) Correlation analysis of AEP expression with the nuclear DDX3X-positive ratio in 2 glioma tissue microarray. Data were plotted as the mean ± SEM. Statistical analysis was performed using an unpaired *t* test (**I**) and Spearman correlation test (**J**). ***P < 0.001. Data shown are representative of 3 independent experiments.

Domain analysis of DDX3X showed that AEP cleavage of DDX3X at N124 resulted in deletion of the N-terminal NES (Figure 3A), suggesting that the nuclear export function of tDDX3X-C might be affected. With a red fluorescent fusion protein and confocal microscopy detection, DDX3X was found to be exclusively localized in the cytoplasm, while DDX3X with NES mutation or deletion presented an obvious nuclear location (Figure 3B). As expected, tDDX3X-C substantially accumulated in the nucleus but was exported to the cytoplasm when the NES sequence (amino acids_12–21_) was added back to the N-terminus of tDDX3X-C (Figure 3C). Moreover, DDX3X and NES-tDDX3X-C were sequestered in the nucleus after treatment with leptomycin B (LMB), which specifically binds CRM1 (Figure 3, D and E). Then, we explored whether the nuclear localization of tDDX3X-C correlated with the nuclear localization signal (NLS) of DDX3X, additionally, the mutation of 2 NLSs on DDX3X did not alter the nuclear aggregation of tDDX3X-C cells (Figure 3F). We speculated that there may be other NLSs on tDDX3X-C or that tDDX3X-C may translocate to the nucleus in a non-NLS-dependent manner.

For further analysis of the cleavage of DDX3X in vivo, red and green fluorescent proteins were fused to the N-terminus and C-terminus of DDX3X, respectively, and cotransfected with AEP into HeLa cells. The results suggested that recombinant DDX3X displayed diffuse distribution when AEP was cotransfected, and the green fluorescent signal could be detected in the nucleus, in contrast to the results when DDX3X was transfected alone (Figure 3G). These data confirmed that tDDX3X-C produced by AEP aggregates in the nucleus due to the absence of the N-terminal NES.

We further analyzed the correlation of AEP and nuclear DDX3X in glioma tissue microarrays (Figure 3H and Supplemental Figure 4). Although DDX3X was present in both the cytoplasm and nucleus in glioma, intranuclear DDX3X was significantly higher in HGG than in LGG (Figure 3I and Supplemental Table 4). Analysis of the correlation between nuclear DDX3X and AEP expression showed that higher levels of AEP were associated with higher levels of nuclear DDX3X (Figure 3J and Supplemental Table 5). All together, these data verified that tDDX3X-C produced by AEP cleavage aggregates in the nucleus due to the absence of the N-terminal NES and confirmed the positive relationship between AEP and nuclear DDX3X in glioma.

### tDDX3X-C produced by AEP cleavage promotes tumor progression in vitro and in vivo.

**Figure 4:**
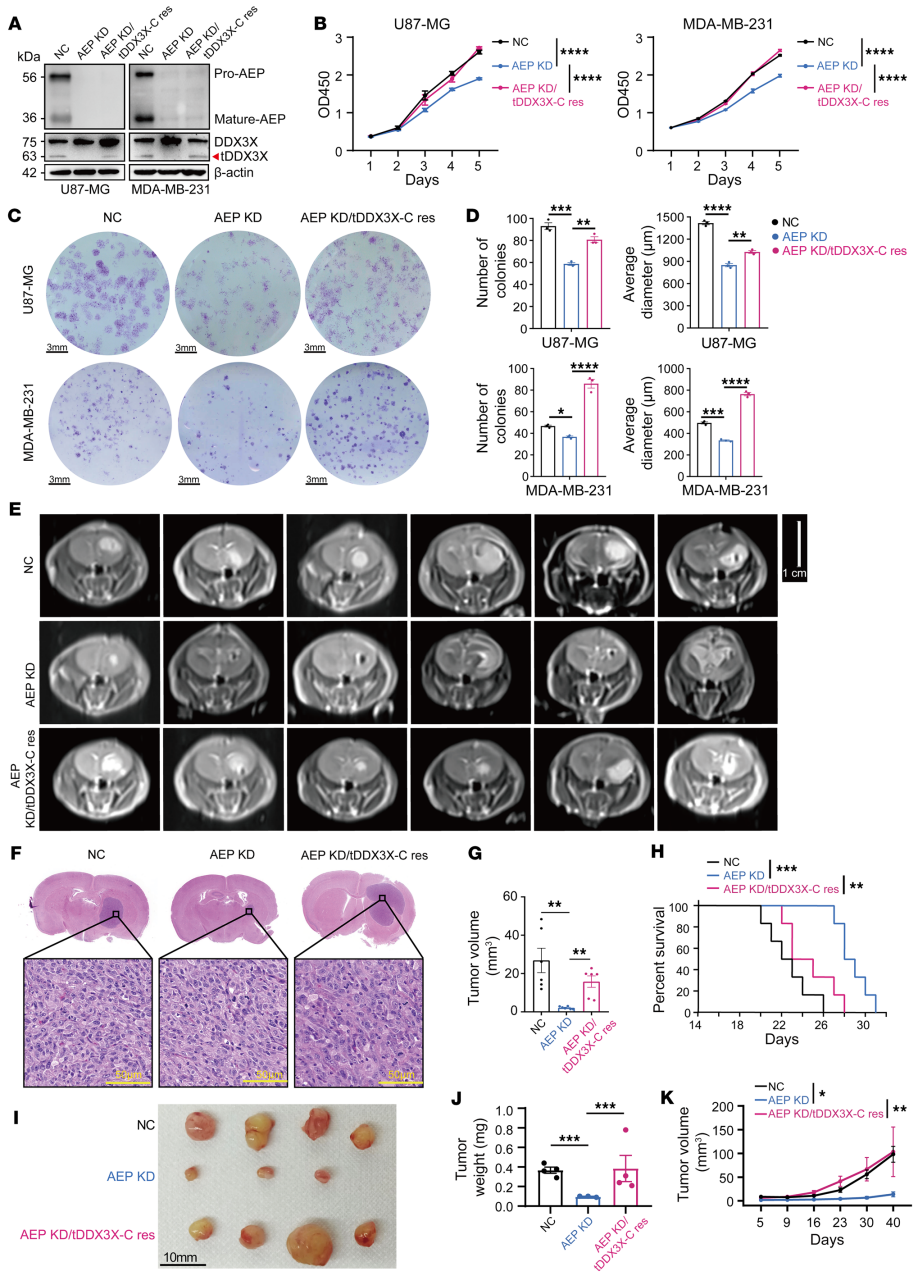
tDDX3X-C produced by AEP promotes tumor progression in vitro and in vivo. (**A**) Immunoblots of AEP, DDX3X, and β-actin in U87-MG and MDA-MB-231 cells in the following groups: the negative control (NC), AEP knockdown (AEP KD) or AEP knockdown, and tDDX3X-C rescue (AEP KD/tDDX3X-C res). (**B**) CCK-8 assays of U87-MG and MDA-MB-231 cells in the groups mentioned in **A**. (**C**) Colony assays of U87-MG and MDA-MB-231 cells in the groups mentioned in **A**. Scale bar: 3 mm. (**D**) The number and average diameter (μm) of the colonies were measured using ImageJ. (**E**) Axial MR images of xenograft glioma generated by orthotopic inoculation of U87-MG cells in the groups mentioned **A**. Scale bar: 1 cm. (**F**) Representative H&E images of orthotopically inoculated glioma grouped by NC, AEP KD, and AEP KD/tDDX3X-C res. Scale bar: 50 μm. (**G**) Tumor volume in mice bearing intracranial glioma from the NC, AEP KD, and AEP KD/tDDX3X-C res groups (*n* = 6). (**H**) Comparative survival of mice bearing intracranial glioma from the indicated groups. (**I**) Morphologic characteristics of xenograft breast cancer models grouped by NC, AEP KD, and AEP KD/tDDX3X-C res (*n* = 4). Scale bar: 10 mm. (**J**) Tumor weight and tumor volume (**K**) of xenograft breast cancer models from the indicated groups. Data were plotted as the mean ± SEM. The time of death in **H** was recorded as the number of days after U87-MG cell implantation. Statistical analysis was performed using 1-way ANOVA followed by Tukey’s multiple comparisons test (**B** and **K**), Šidák’s multiple comparisons test (**D** and **K**), log-rank (Mantel-Cox) test (**H**), and unpaired t test (**G** and **J**). *P < 0.05, **P < 0.01, ***P < 0.001, ****P < 0.0001. Data shown are representative of 3 independent experiments.

To explore the effect of AEP cleavage of DDX3X on cancer progression, we constructed glioma and breast cancer cells (U87-MG, U251-MG, and MDA-MB-231) with AEP KD as well as with tDDX3X-C rescue (AEP KD/tDDX3X-C res) and verified the lines (Figures 4A and Supplemental Figure 5A). Cells with AEP KD showed impaired proliferation, which was consistent with our previous findings (14, 15, 17). However, the rescue of tDDX3X-C strikingly enhanced the proliferation and colony formation of AEP KD cells (Figure 4, B–D and Supplemental Figure 5, B–D). Additionally, we assessed the impact of the DDX3X N124A site on the proliferative phenotype of tumor cells by soft agar colony formation assays, grouped as the negative control (NC), DDX3X KD, DDX3X KD/DDX3X WT res, and DDX3X KD/DDX3X N124A res groups. As shown in Supplemental Figure 5, E–H, DDX3X KD significantly inhibited the proliferative capacity of tumor cells, while rescue with WT DDX3X effectively alleviated this impairment in proliferation. However, the N124A site mutant did not exhibit the same rescue capability.

To fully address the effects of AEP cleavage of DDX3X on cancer progression in vivo, we used glioma and breast cancer orthotopic tumor models using NC, AEP KD, and AEP KD/tDDX3X-C res U87-MG or MDA-MB-231 cells. Consistent with previous studies, AEP KD significantly inhibited tumor development, while tDDX3X-C rescue promoted tumor progression of AEP-KD cells both in U87-MG (Figure 4, E–G) and MDA-MB-231 cells (Figure 4, I–K and Supplemental Figure 6A). The results from the survival analysis of 3 groups of mice bearing glioma showed that AEP KD significantly prolonged overall survival (OS) from 23 to 28 days (*P* = 0.0005), while tDDX3X-C rescue in turn led to worsening and shortening of survival in the AEP KD group of mice (*P* = 0.0076) (Figure 4H). Furthermore, the proliferative abilities of these tumors were confirmed by IHC staining of Ki-67 (Supplemental Figure 6, B–E). These results suggested that DDX3X-C produced by AEP cleavage is important for cancer progression, especially tumor proliferation.

### AEP promotes oncogenic splicing of widespread pre-mRNAs via tDDX3X-C, especially exon skipping of PRDM2 exon 2 and ARRB1 exon 13.

**Figure 5:**
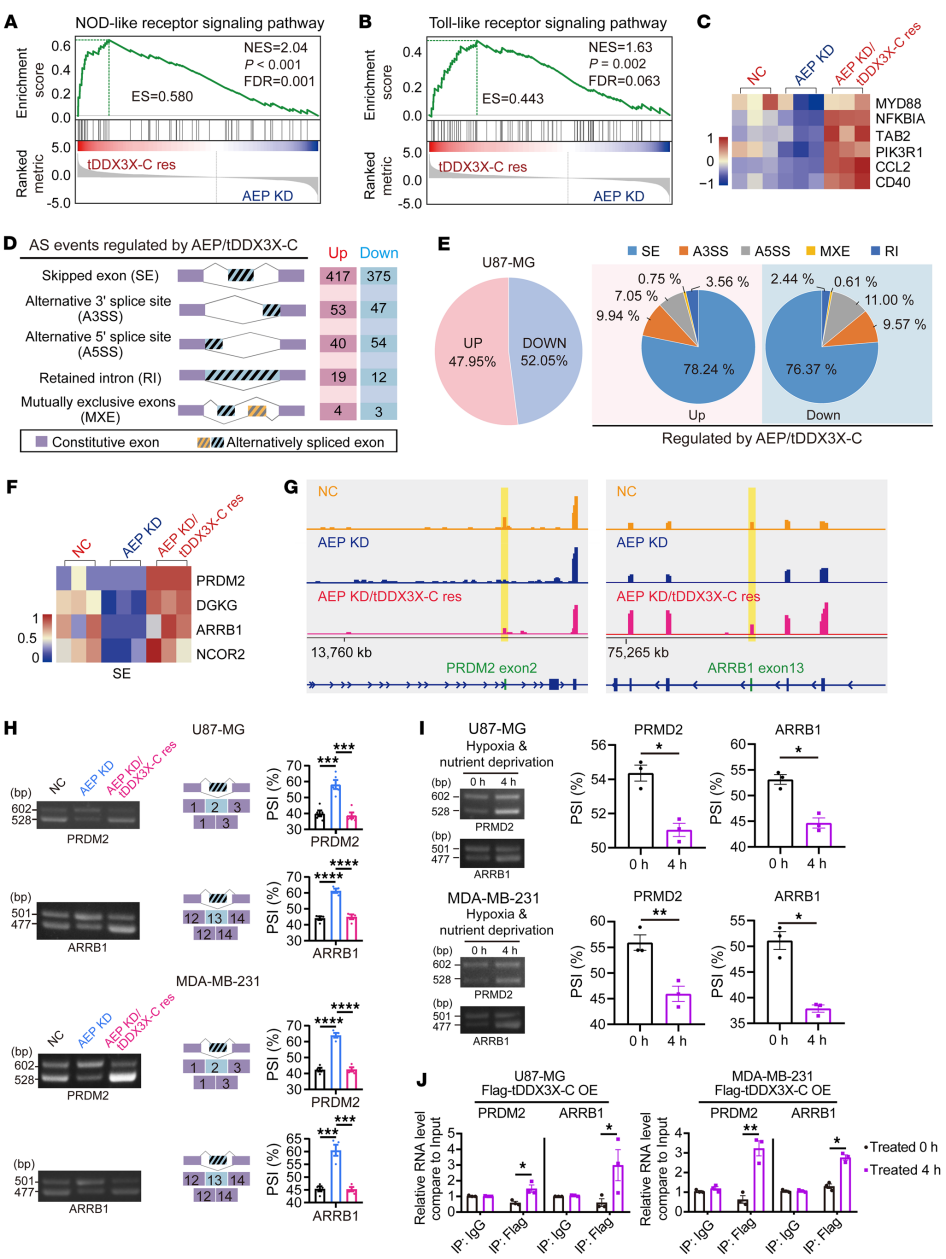
AEP promotes oncogenic splicing of widespread pre-mRNAs via tDDX3X-C. (**A** and **B**) GSEA of the expression profile of the NOD-like signaling pathway and Toll-like receptor signaling pathway in U87-MG cells (AEP KD or AEP KD/tDDX3X-C res). (**C**) Heatmap for gene sets of the Toll-like signaling pathway and the NOD-like signaling pathway regulated by AEP/tDDX3X-C. (**D**) Summary of differential splicing analysis performed using U87-MG cells in the following groups: NC, AEP KD, and AEP KD/tDDX3X-C res. The numbers of identified AS events in each category upon AEP/tDDX3X-C regulation are indicated. (**E**) Pie charts showing the percentages of changed AS events identified in U87-MG cells (left); pie charts representing the distribution of AEP/tDDX3X-C-regulated splicing events among different splicing profiles (right). (**F**) Representative heatmap for changed AS events from skipped exons (SEs) positively regulated by AEP/tDDX3X. (**G**) RNA-Seq results of alternative sites in the indicated genes using IGV software analysis. (**H**) PCR and AGE analyses for *PRDM2* exon 2 and *ARRB1* exon 13 regulated by AEP/tDDX3X-C in U87-MG and MDA-MB-231 cells. The middle panels show schematic diagrams of the indicated AS exons. Right panels show the quantification of percent spliced in (PSI). (**I**) PCR and AGE analyses for *PRDM2* exon 2 and *ARRB1* exon 13 in U87-MG and MDA-MB-231 cells with or without hypoxia and nutrient deprivation stimulus for 4 hours. (**J**) RNA-IP results of U87-MG and MDA-MB-231 cells (Flagged-tDDX3X-C OE) with or without hypoxia and nutrient deprivation stimulus for 4 hours. Data were plotted as the mean ± SEM. Statistical analysis was performed using 1-way ANOVA followed by Tukey’s multiple comparisons test (**H**), paired *t* test (**I**), and unpaired *t* test (**J**). *P < 0.05, **P < 0.01, *** *P <* 0.001, ****P < 0.0001. Data shown are representative of 3 independent experiments.

As the DDX family participates in RNA metabolism, especially transcription and splicing, we performed RNA-Seq of U87-MG cells with AEP KD, AEP KD/tDDX3X rescue or the NC and determined the AEP/tDDX3X-driven transcriptome. We identified 112 differentially expressed genes (DEGs) (Supplemental Table 6) by using a 2.0-fold cut-off and *q* value of less than 0.05 threshold for inclusion. Gene set enrichment analysis (GSEA) revealed that AEP/tDDX3X-C enhanced NOD-like and Toll-like receptor signaling activation by regulating key factors in this pathway (Figure 5, A–C).

To gain insight into the profile of RNA splicing, we used rMATS software to perform differential exon usage analysis based on our RNA-Seq data. The results of the differential exon analysis suggested that AEP/tDDX3X-C regulate multiple types of AS of widespread pre-mRNAs, including exon skipping (ES), alternative 3′ splice sites (A3SSs), alternative 5′ splice sites (A5SSs), mutually exclusive exons (MXEs) and retained introns (RIs), and ES was the most common (Figure 5, D and E and Supplemental Table 7). We identified exons with significant loss or gain among the 3 groups with a FDR thresholds of 0.05 and an IncLevelDifference absolute value greater than 0.1 (Supplemental Figure 7A). The changes in A3SSs are also shown in Supplemental Figure 7B.

To further verify these AS events, we selected the tumor suppressor genes PR/SET domain 2 (PRDM2), diacylglycerol kinase gamma (DGKG), β-arrestin 1 (ARRB1), and nuclear receptor corepressor 2 (NCOR2) (Figure 5, F and G and Supplemental Figure 7C). PCR and subsequent agarose gel electrophoresis (AGE) results showed that AEP/tDDX3X-C enhanced the ES of *PRDM2* exon 2, *ARRB1* exon 13, and *NCOR2* exon 21 in both glioma and breast cancer cells (Figure 5H and Supplemental Figure 7D). U87-MG and MDA-MB-231 cells treated with hypoxia and nutrient deprivation for 4 hours showed a significant enhancement of the specific AS of *PRDM2* and *ARRB1* (Figure 5I). Moreover, RNA IP with tDDX3X-C identified significant binding of *PRDM2* and *ARRB1* mRNAs after hypoxia and nutrient deprivation (Figure 5J). Additionally, sequencing of the PCR product confirmed the ES sites of *PRDM2* and *ARRB1* (Supplemental Figure 7E).

### tDDX3X-C interacts with splicing factors and regulates AS in a manner partially dependent on hnRNPA1.

**Figure 6:**
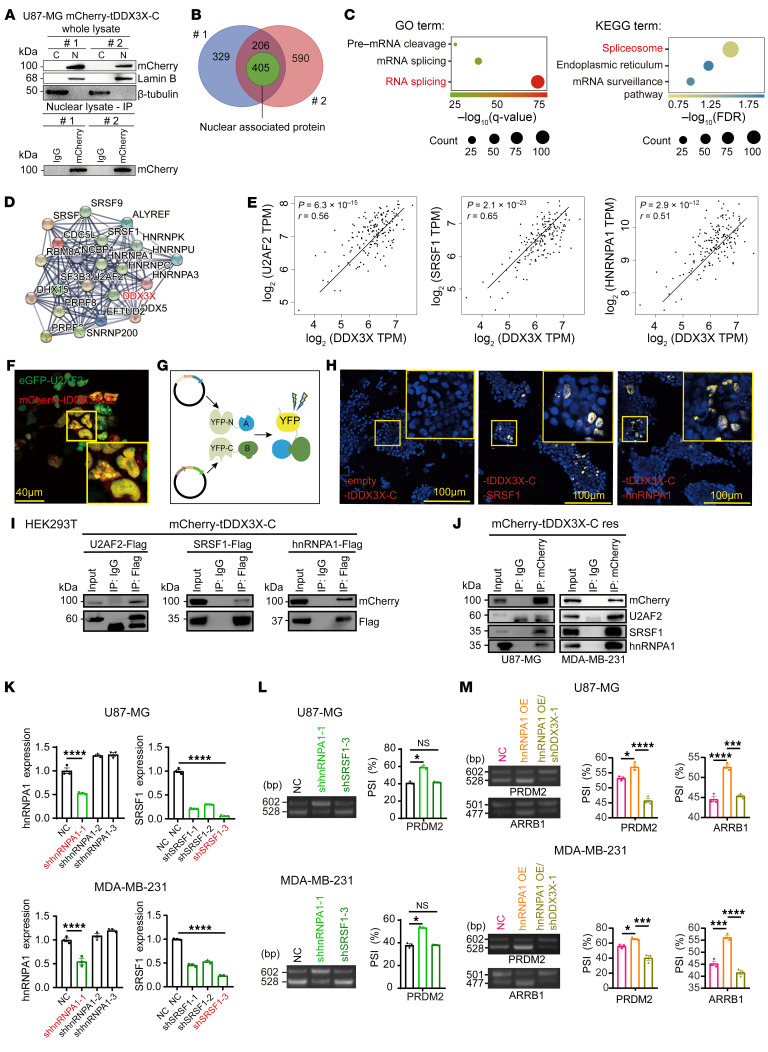
tDDX3X-C interacts with spliceosome factors and regulates AS in a manner partially dependent on hnRNAPA1. (**A**) Immunoblots of mCherry-tagged tDDX3X-C, lamin B and β-tubulin in the nuclear and cytoplasmic fractions of U87-MG-mCherry-tDDX3X-C cells. mCherry antibody (1:50) was used to precipitate mCherry-tDDX3X-C in the nuclear lysate of 2 cell samples; #1, sample 1; #2, sample 2; C, cytoplasmic; N, nuclear. (**B**) Venn diagram showing the intersection of 2 sets of proteins detected by IP-MS. There were 611 kinds of proteins in the intersection and 405 kinds of nuclear proteins are represented in green circle. (**C**) GO and KEGG term enrichment analyses of the DDX3X-interacting nuclear proteins identified from the IP-MS data. (**D**) String 10.5 program (http://string-db.org) analyses showing the interaction networks among 21 candidate proteins associated with mRNA splicing. (**E**) Expression correlation analysis of DDX3X and splicing factors based on the TCGA database. (**F**) HEK293T cells were cotransfected with eGFP-U2AF2/mCherry-tDDX3X-C. Scale bar: 40 μm. (**G**) Diagram of BIFC. (**H**) HEK293T cells were transfected with YN155-empty vector/YC156-tDDX3X-C, YN155-SRSF1/YC156-tDDX3X-C, or YN155-hnRNPA1/YC156-tDDX3X-C. Scale bar: 100 μm. (**I**) Co-IP assays of HEK293T cells transfected with the indicated plasmids. (**J**) U87-MG and MDA-MB-231 cells expressing tDDX3X-C (1 × 10^7^) were harvested for Co-IP assays. (**K**) RT-qPCR assays of the indicated mRNAs in U87-MG and MDA-MB-231 cells. (**L**) PCR and AGE analyses for *PRDM2* exon 2 in U87-MG and MDA-MB-231 in the following groups: NC, shSRSF1-3 and shhnRNPA1-1; quantification of percent spliced in (PSI). (**M**) PCR and AGE analyses for *PRDM2* exon 2 and *ARRB1* exon 13 in U87-MG and MDA-MB-231 grouped by NC, hnRNPA1 OE, and hnRNPA1 OE/shDDX3X-C-1. Data were plotted as the mean ± SEM. Statistical analysis was performed using 1-way ANOVA followed by Dunnett’s multiple comparisons test (**K** and **L**) and Tukey’s multiple comparisons test (**M**). *P < 0.05, ***P < 0.001, ****P < 0.0001. Data shown are representative of 3 independent experiments.

To clarify the molecular mechanisms of tDDX3X-C in the nucleus, we isolated the nuclear fractions of mCherry-tDDX3X-C overexpressing U87-MG cells using a nucleoplasmic separation assay. Consistent with Figure 3B, tDDX3X-C abundantly accumulated in the nucleus (Figure 6A). Next, nuclear tDDX3X-C interactions were detected by nuclear fraction IP/MS using an anti-mCherry antibody (Figure 6B and Supplemental Table 8). Gene Ontology (GO) and Kyoto Encyclopedia of Genes and Genomes (KEGG) pathway enrichment analyses suggested that tDDX3X-C complexed with proteins associated with pre-mRNA cleavage and mRNA and RNA splicing, among which large amounts of proteins were involved in spliceosome-mediating RNA splicing processes (Figure 6C). Protein-protein interaction (PPI) network analysis (36) demonstrated the interaction of DDX3X with many splicing-related proteins (Figure 6D). Moreover, expression correlation analysis suggested that DDX3X was positively associated with splicing factors (Figure 6E and Supplemental Figure 8), suggesting that DDX3X may directly participate in AS regulation. We fused eGFP to the spliceosome component U2 auxiliary factor 65 kDa subunit (eGFP-U2AF2) and cotransfected eGFP-U2AF2 with mCherry-tDD3X3-C into HEK293T cells; the results showed that both were enriched in the nucleus and had significant colocalization (Figure 6F).

In addition to the spliceosome components, 2 major regulatory protein families are involved in modulating the splicing reaction and act as splicing activators or repressors by binding to exonic or intronic enhancer or silencer elements. Serine/arginine rich splicing factor 1 (SRSF1) and heterogeneous nuclear ribonucleoprotein A1 (hnRNPA1) are representative of these 2 families. Bimolecular fluorescence complementation (BiFC) analysis confirmed the close interactions of tDDX3X-C and SRSF1 or hnRNPA1 (Figure 6, G and H). Moreover, endogenous and exogenous co-IP experiments were applied to investigate the interaction of tDDX3X-C with U2AF2, SRSF1, and hnRNPA1 (Figure 6, I and J). Although SRSF1 and hnRNPA1 are both frequently upregulated in cancer, they have opposite functions in AS events (37). To further clarify this difference, we knocked down SRSF1 and hnRNPA1. Efficient KD of hnRNPA1 significantly suppressed the ES of *PRDM2* exon 2, while KD of SRSF1 did not have a significant effect (Figure 6, K and L). To establish the dependency of hnRNPA1 on tDDX3X-C for its splicing function, we carried out an experiment where hnRNPA1 was overexpressed in cells lacking DDX3X. As shown in Figure 6M, AS of *PRDM2* and *ARRB1* were elevated after hnRNPA1 overexpression. However, in hnRNPA1 OE/DDX3X KD cells, AS of *PRDM2* and *ARRB1* did not increase. Thus, these results indicate that tDDX3X-C is highly complexed with spliceosome factors in the nucleus and regulates AS in a manner partially dependent on hnRNPA1.

### AEP/tDDX3x–induced PRDM2-Δexon 2 and ARRB1-Δexon 13 promote tumor malignancy.

To determine the effects of PRDM2-Δexon 2 and ARRB1-Δexon 13 isoforms on cancer cell function, we performed rescue experiments using the following groups of cancer cells: NC, AEP KD, AEP KD/tDDX3X-C res, AEP KD/PRDM2 KD, and AEP KD/ARRB1-Δexon 13 res cells (Supplemental Figure 9, A and B). Proliferation-related assays, including Cell Counting Kit-8 (CCK-8) and colony formation assays, were performed, and as expected, loss of PRDM2 or gain of the ARRB1-Δexon 13 isoform induced by AEP/tDDX3X-C promoted cancer cell proliferation (Supplemental Figure 9, C–F).

**Figure 7:**
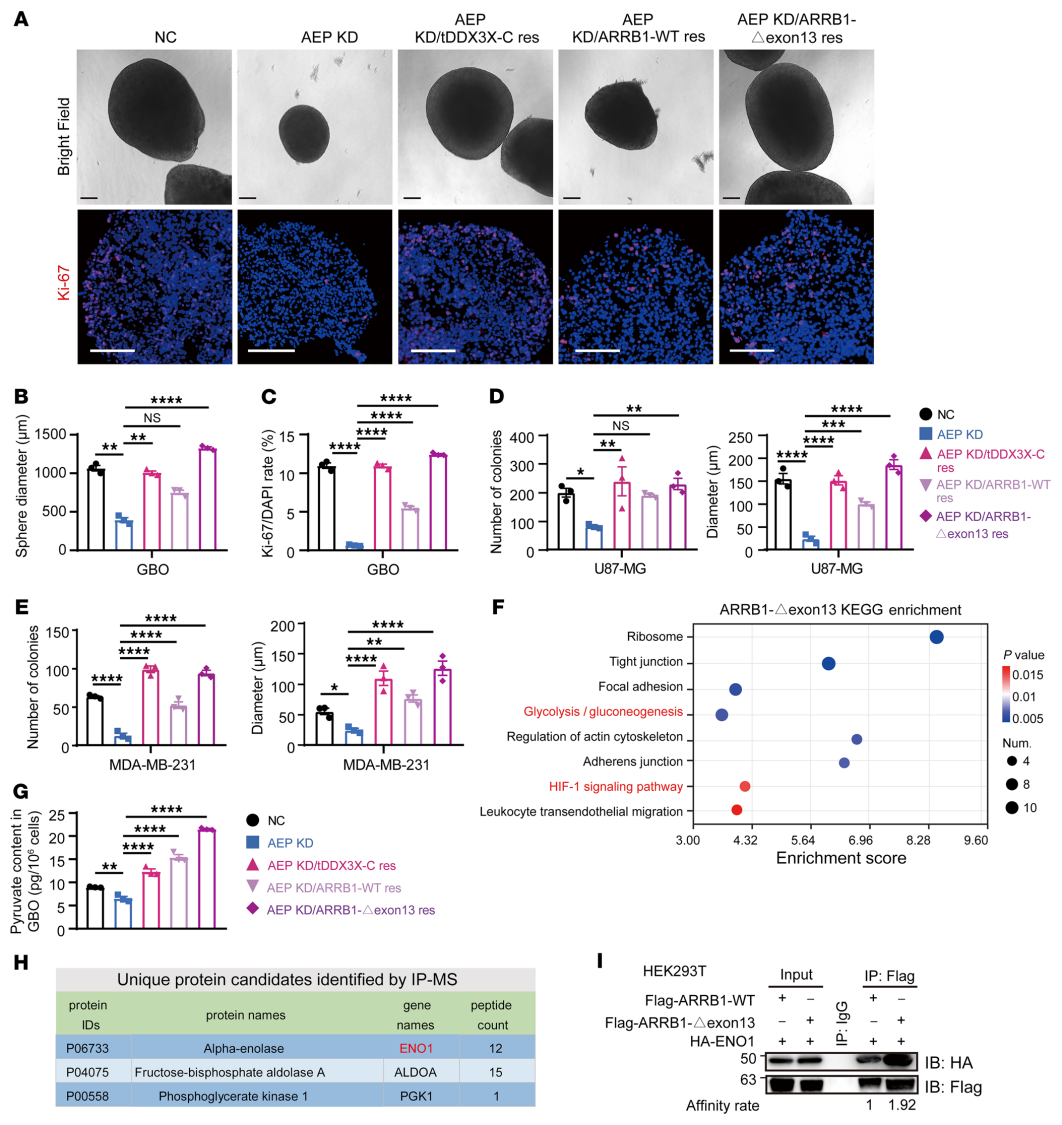
ARRB-Δexon 13 proteoform is critical for AEP/tDDX3X-C-mediated sustained tumor growth. (**A**) Representative bright field images of GBO no. 1 and the corresponding IF of Ki-67 were used to detect the viability of GBOs. Scale bar: 200 μm. (**B**) Histogram of the diameter of GBOs. (**C**) Ratio of Ki-67 in GBOs. (**D** and **E**) Histogram of the number and diameter of colonies in soft agar colony formation assays of U87-MG (**D**) and MDA-MB-231 (**E**) cells in the NC, AEP KD, AEP KD/tDDX3X-C res, AEP KD/ARRB1-WT res, and AEP KD/ARRB1-Δexon 13 res groups. The number and average diameter of cell colonies (μm) were counted and measured by ImageJ. (**F**) Protein binding profile of ARRB1-Δexon 13 detected by IP-MS. (**G**) Pyruvate content in the corresponding groups of GBOs. (**H**) Preferential binding proteins of ARRB1-Δexon 13 associated with glycolysis. (**I**) Co-IP assays of ARRB1-Δexon 13, ARRB1-WT, and ENO1. Data were plotted as the mean ± SEM, and statistical analysis was performed using 1-way ANOVA followed by Šidák’s multiple comparisons test. *P < 0.05, **P < 0.01, ***P < 0.001, ****P < 0.0001. Data shown are representative of 3 independent experiments.

As patient-derived GBM organoids (GBOs) recapitulate the histological features of GBM (38), we successfully established GBOs (Supplemental Figure 10A) and used molecular profiling to validate the consistency between GBOs and GBM tissues (Supplemental Table 9). AEP was highly expressed in the region outside the necrotic area (Supplemental Figure 10B), which is consistent with our findings that hypoxia/nutrient deficiency triggered AEP. Furthermore, colocalization of AEP and DDX3X was observed in the cytoplasm in frozen tissue sections of GBOs and in HeLa cells (Supplemental Figure 10C). We then performed rescue experiments in various types of GBOs: NC, AEP KD, AEP KD/tDDX3X-C res, AEP KD/ ARRB1-WT res, and AEP KD/ARRB1-Δexon 13 res (Supplemental Figure 10, D and E). Microscopic observation and Ki-67 staining results showed that gain of the ARRB1-Δexon 13 isoform induced by AEP/tDDX3X-C more significantly promoted GBO growth than that of the ARRB1-WT isoform (Figure 7, A–C and Supplemental Figure 11, A and B). Soft agar colony assays were also performed. Consistent with the literature, which reported promotion of tumor proliferation in gastric cancer (39), WT ARRB1 could partially rescue the AEP KD phenomenon in GBM and breast cancer. However, gain of the ARRB1-Δexon 13 isoform resulted in a stronger tumor-promoting capacity than that of the WT ARRB1 (Figure 7, D and E and Supplemental Figure 11C).

To clarify the mechanisms of ARRB1-Δexon 13, we immunoprecipitated ARRB1-Δexon 13 and performed a mass spectrum assay. KEGG analysis revealed that unlike ARRB1-WT, ARRB1-Δexon 13 binds to proteins involved in glycolysis and the HIF-1 signaling pathway (Figure 7F, Supplemental Figure 11D, and Supplemental Table 10). Pyruvate content was decreased significantly after AEP knockdown, while gain of tDDX3X-C or the ARRB1-Δexon 13 isoform significantly increased its content in GBOs. ARRB1-WT also partially rescued the pyruvate content (Figure 7G and Supplemental Figure 11E). Glycolytic enzymes, including ENO1, ALDOA, and PGK1, which are associated with glycolysis, interacted with ARRB1-Δexon 13 (Figure 7H). Co-IP experiments confirmed the interaction between ARRB1-Δexon 13 and ENO1 (Figure 7I). Thus, AEP promoted oncogenic splicing of widespread pre-mRNAs via tDDX3X-C, and the PRDM2-Δexon 2 and ARRB1-Δexon 13 isoforms induced by AEP/tDDX3x promote cancer cell proliferation.

### AEP/tDDX3X/ARRB1-Δexon 13 is highly expressed in cancerous tissues and is tightly associated with poor patient prognosis.

**Figure 8:**
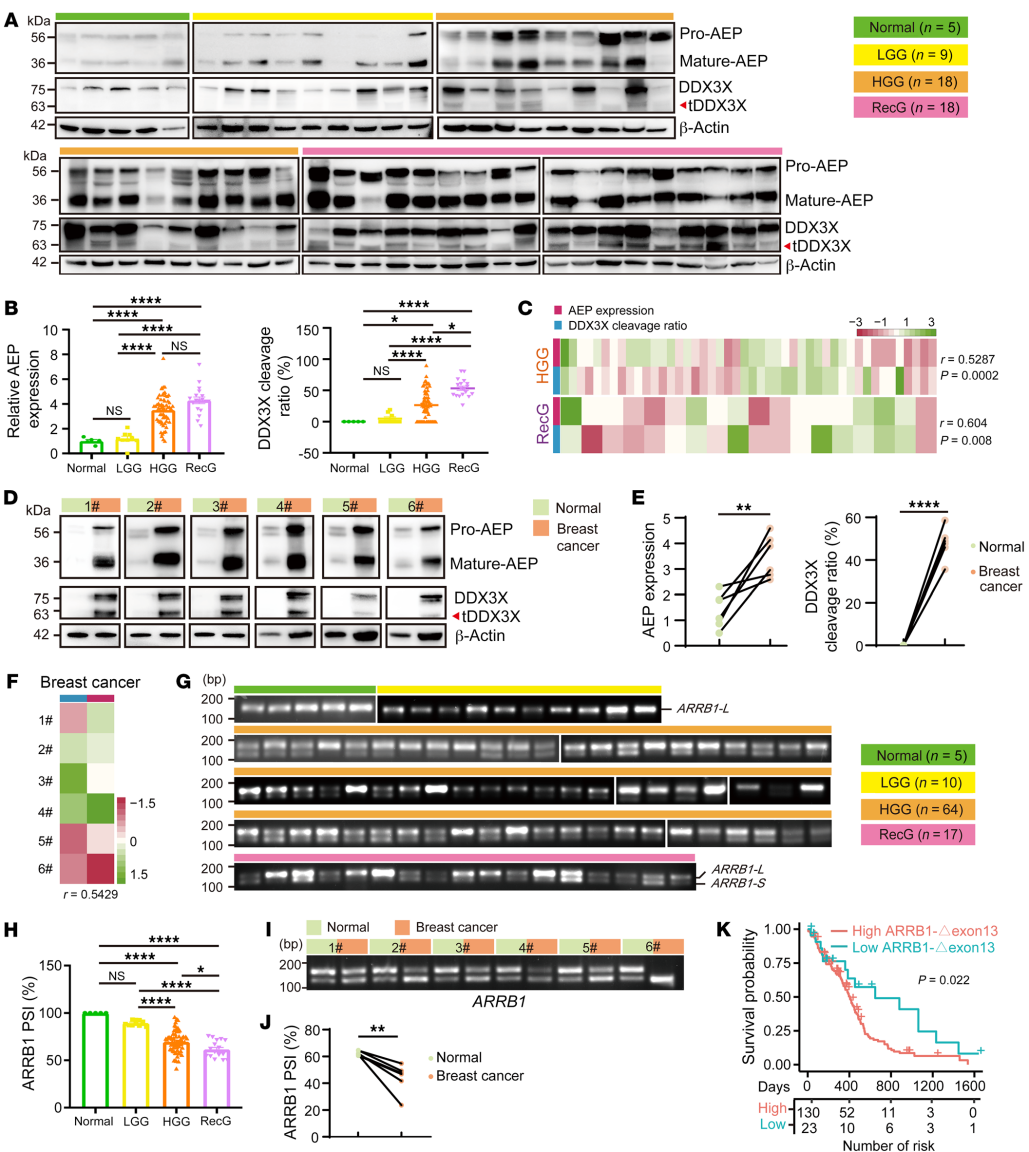
AEP/tDDX3X-mediated *ARRB1-*Δ*exon 13* splicing was highly prevalent in cancerous tissues and tightly associated with poor patient prognosis. (**A**) Immunoblots of AEP, DDX3X and β-actin in normal brain tissues (*n* = 5) and glioma cancerous tissues, including LGG (*n* = 9), HGG (*n* = 18), and RecG (*n* = 18). (**B**) Quantification of AEP expression (left panel) and the DDX3X cleavage ratio (tDDX3X-C/DDX3X, right panel) in a group of tissues based on the immunoblot results. (**C**) Correlation analysis of AEP expression and the DDX3X cleavage ratio in HGG and RecG tissues. (**D**) Immunoblots of AEP, DDX3X, and β-actin in 6 pairs of breast cancer and adjacent normal tissues. (**E**) Quantification of AEP expression and the DDX3X cleavage ratio in **D**. (**F**) Correlation analysis of AEP expression and the DDX3X cleavage ratio in 6 breast cancerous tissues. (**G**) PCR and AGE analyses for *ARRB1-WT and ARRB1-*Δ*exon 13* in normal brain tissues (*n* = 5) and glioma cancerous tissues, including LGG (*n* = 10), HGG (*n* = 64), and RecG (*n* = 17). (**H**) Quantification of the PSI based on **G**. (**I**) PCR and AGE analyses of *ARRB1-WT and ARRB1-*Δ*exon 13* in 6 pairs of breast normal tissues and cancerous tissues. (**J**) Quantification of the PSI in breast tissues based on **I**. (**K**) The prognostic significance of *ARRB1-*Δ*exon 13* in GBM patients is shown in TCGA data sets. Data were plotted as the mean ± SEM, and statistical analysis was performed using 1-way ANOVA followed by Tukey’s multiple comparisons test (**B** and **H**) and paired *t* test (**E** and **J**). The Spearman correlation test was considered to indicate statistical significance in (**C** and **F**). Heatmaps (**C** and, **F**) were drawn by the online website Heatmapper (www.heatmapper.ca). *P < 0.05, **P < 0.001, ****P < 0.0001.

To further clarify the pathological importance of AEP/tDDX3X–mediated *ARRB1-*Δ*exon 13* splicing in cancer, we performed Western blotting and verified the significantly higher protein levels of AEP and tDDX3X in HGG and recurrent glioma (RecG) than in normal tissues and LGG (Figure 8, A–C and Supplemental Figure 12A). Significant positive correlations between AEP expression and the DDX3X cleavage ratio were found (Figure 8C). Consistently, the levels of AEP and tDDX3X were much higher in breast cancer tissues than in normal tissues, and the expression levels of both showed a positive correlation. (Figure 8, D–F). Moreover, the levels of *ARRB1-*Δ*exon 13* in HGG and RecG tissues and breast cancer tissues were analyzed, and we found that this alternative isoform, *ARRB1-*Δ*exon 13*, was upregulated in cancerous tissues compared with normal tissues and LGG (Figure 8, G–J). Significant negative correlations between *ARRB1-*Δ*exon 13* and AEP expression or the DDX3X cleavage ratio were found (Supplemental Figure 12B). High *NCOR2-*Δ*exon 21* levels were also found in HGG (Supplemental Figure 12, C and D). Furthermore, the TCGA database was analyzed, and we found that high *ARRB1-*Δ*exon 13* expression was associated with a poor prognosis in patients with GBM (Figure 8K). Consistent with low *PRDM2-*Δ*exon 2*, the expression of PRDM2 was much lower in cancerous tissues than in normal controls (Supplemental Figure 12E). The results of snRNA-Seq of 6 cases of GBM also support that *PRDM2* was expressed at low levels in most clusters (Supplemental Figure 12F). Thus, these results indicate that AEP/tDDX3X-mediated *ARRB1-*Δ*exon 13* splicing is prevalent in cancerous tissues and is tightly associated with a poor patient prognosis. Taken together, these data suggest that AEP-specific cleavage of DDX3X leads to nuclear translocation and that nuclear tDDX3X-C regulates oncogenic splicing in multiple kinds of malignant tumors, especially via *PRDM2* and *ARRB1*, under tumor microenvironmental stimuli dependent on HIF1A.

## Discussion

In this study, we highlight a molecular mechanism for cancer cell adaptation to harsh microenvironments in various kinds of solid tumors. Hypoxia and nutrient deficiency trigger a protease, AEP, thus inducing precise cleavage of DDX3X, which dysregulates AS in a HIF1A-dependent manner, indicating that tumor microenvironment–regulated proteomic and transcriptomic heterogeneity can be achieved by precise protease substrate cleavage without genetic/epigenetic changes. Protease-specific hydrolysis of substrate proteins is essentially a special type of protein posttranslational modification (40). In recent years, the importance of protease-specific regulation has been recognized (41, 42). In this study, we revealed a mechanism by which cancer cells convert tumor microenvironmental stimuli to nuclear AS changes, illuminating the crucial role of the protease cleavage regulatory network in tumor malignancy.

Proteolytic cleavage affects protein structure and subcellular localization and yields novel proteoforms that execute new functions (43). The present study shows that truncated DDX3X induced by AEP accumulates in the nucleus, and, functionally, it is different from full-length DDX3X. tDDX3X-C in the nucleus is located in the spliceosome and is mainly involved in pre-mRNA AS. In fact, the generation of proteoforms through proteolytic cleavage is a widespread intracellular posttranscriptional modification mechanism with diverse important physiological and pathological functions (44). Similar to the effects of DDX3X cleavage, cleavage of amyloid-β precursor protein (APP) by AEP results in truncated APP (C586–689), which binds to CCAAT enhancer binding protein β (CEBPB), leading to its nuclear translocation and promoting AD progression (45). Likewise, Tmod3-C translocates to the nucleus and affects transcription (17). Truncations produced by AEP-targeted cleavage may exert an important regulatory effect. More importantly, we found a regulated pattern in which the AEP cleavage site was primarily located at the IDR, which can preserve the intact functional domains of substrates, indicating its important role in precision proteolysis and regulatory proteolytic events. Narrow cleavage specificity combined with clear cleavage preference for unstructured secondary regions in substrates has been reported (46). Additionally, ELM analysis revealed multiple potential modification sites within the DDX3X 1–124 region, such as S58–K64, which may undergo CK1 phosphorylation; G80–F85, which may undergo NEK2 phosphorylation, and G89–S92, which may serve as a glycosaminoglycan attachment site. These diverse modifications might potentially influence the efficiency of AEP cleavage of DDX3X and need further investigation. However, the precise regulatory mechanisms of IDRs remain largely unknown. In particular, the tumor intrinsic stresses that trigger precise modifications of the IDR need further study.

DDX3X was found to be one of the core factors of RNA splicing, participating in the formation of affinity-purified human spliceosomes (47), the spliceosome B complex (48), and messenger ribonucleoprotein (mRNP) binding (49). Moreover, DDX3X suppresses KLF4 expression by manipulating KLF4 mRNA AS (12). However, whether AEP explicitly cleaves DDX3X is unclear, and the pathophysiological importance of DDX3X cleavage remains to be determined. Our endogenous IP/MS and biochemical experiments revealed that DDX3X is a specific target of AEP triggered by hypoxia and nutrient deficiency. Especially in glioma, higher levels of AEP were associated with higher levels of nuclear DDX3X, which supports the potential pathological importance of AEP cleavage-induced nuclear DDX3X. Moreover, AEP-targeted cleavage of DDX3X at N124 produced nuclear tDDX3X-C–regulated oncogenic splicing of widespread pre-mRNAs. Through further MS analysis, we confirmed that hnRNPA1 is an important factor involved in this process. As a splicing factor, hnRNPA1 has been found to be influenced by hypoxia (12), arginine methylation (50), and its interaction with other proteins, such as PHB2 (51). However, whether the interaction between tDDX3X-C and hnRNPA1 influences the functions of hnRNPA1 or the activity of spliceosomes requires further analysis. AEP/tDDX3X-C–mediated AS events as well as produced isoforms are highly associated with cancer malignancy and poor prognosis, and, thus, may be potential biomarkers or therapeutic targets for cancer. PRDM2 or RIZ1 is a tumor suppressor gene and a member of a nuclear histone methyltransferase superfamily that encodes a zinc finger protein that can bind to retinoblastoma protein, oestrogen receptor, and the TPA-responsive element (MTE) of the haem oxygenase-1 gene (52). Although genetic inactivation, epigenetic silencing, chromosomal deletion, or alternative use of different promoters has been frequently revealed in several cancers (53, 54), the AS event of PRDM2 has not been reported. AS of PRDM3 has been found in myeloid leukemia (55). In this study, a frameshift of the tumor suppressor PRDM2 caused by AEP/tDDX3X-C–regulated skipping of exon 2 was found.

Moreover, ARRB1-Δexon 13 isoform was verified to have oncogenic functions and was associated with a poor prognosis. ARRB1 is a ubiquitously expressed scaffold protein that negatively regulates G protein-coupled receptor (GPCR) signaling (56). β-arrestins regulate mitogenic and antiapoptotic signaling and play an essential role in tumor vascularization, metastasis, and cancer stem cell maintenance (56–58). Two alternatively spliced isoforms of human ARRB1, differing only in the presence or absence of 24 base pairs/8 amino acids (exon 13) within the sequence, were identified with unknown origin and functions (59). More interestingly, ARRB1-Δexon 13–enhanced malignant growth of cancer cells is associated with glycolysis and the HIF-1 signaling pathway. The oncogenic functions and mechanisms of ARRB1-Δexon 13 need further study.

In conclusion, our study reveals the interaction between AEP and DDX3X, which provides a new layer of knowledge regarding protease-mediated regulation of AS in the harsh tumor microenvironment. We believe that our findings elucidated the mechanisms of tumor cell adaptation to the evolving tumor microenvironment during tumor development through delivery of external stimuli to the center nucleus. These findings might offer critical insights for cancer diagnosis and treatment by targeting proteases and their downstream specific proteoforms that could be potential diagnostic/therapeutic targets.

## Methods

### Cell lines.

The human embryonic kidney cell line (HEK293T) and tumor cell lines U87-MG, A172 (human glioma), MAD-MB-231, MDA-MG-468, BT20, MCF7, ZR-75-1, BT-474, SKBR3, MDA-MB-435S, AU565 (human breast cancer cells), PANC-1 (human pancreatic cancer cells), and 143B (human osteosarcoma cells) were obtained from the American Type Culture Collection (ATCC). The tumor cell lines HeLa (human cervical cancer cells) and U251-MG (human astrocytoma cells) were purchased from the National Collection of Authenticated Cell Cultures. Detailed identifiers are listed below for human HEK293T (ATCC, Cat CRL-3216, RRID: CVCL_0063), human U87-MG (ATCC, Cat HTB-14, RRID: CVCL_0022), human A172 (ATCC, Cat CRL-7899, RRID: CVCL_0131), human MDA-MB-231 (ATCC, Cat CRM-HTB-26, RRID: CVCL_0062), human MDA-MB-468 (ATCC, Cat HTB-132, RRID: CVCL_0419), human BT20 (ATCC, Cat HTB-19, RRID: CVCL_0178), human MCF7 (ATCC, Cat HTB-22, RRID: CVCL_0031), human ZR-75-1 (ATCC, Cat CRL-1500, RRID: CVCL_0588), human BT-474 (ATCC, Cat HTB-20, RRID: CVCL_0179), human SKBR3 (ATCC, Cat HTB-30, RRID: CVCL_0033), human MDA-MB-435S (ATCC, Cat HTB-129, RRID: CVCL_0622), human AU565 (ATCC, Cat CRL-2351, RRID: CVCL_1074), human PANC-1 (ATCC, Cat CRL-1469, RRID: CVCL_0480), human 143B (ATCC, Cat CRL-8303, RRID: CVCL_2270), human U251-MG (National Collection of Authenticated Cell Cultures, Cat TCHu58, RRID: CVCL_0021), human HeLa (National Collection of Authenticated Cell Cultures, Cat TCHu187, RRID: CVCL_0030), and human SGC-7901 (Donated by Department of Digestive Surgery).

All cells were maintained in MEM, DMEM, or RPMI-1640 (ll from HyClone) containing 10% FBS (Gibco) and 100 IU/mL penicillin/streptomycin (Solarbio) at 37°C in a humidified incubator with 5% CO_2_.

### Western blotting.

Cell lysates as indicated were prepared in RIPA buffer (Solarbio) containing phenylmethylsulfonyl fluoride (Sigma-Aldrich) and complete protease inhibitor cocktail (Roche). Lysates were incubated for 30 minutes on ice and centrifuged for 10 minutes at 12,500*g* at 4°C. A BCA protein assay kit (Thermo Fisher Scientific) was used to assay the protein concentrations. Samples were separated by SDS-PAGE and transferred to PVDF membranes. After the membranes were blocked in TBST (TBS with 0.1% Tween-20) containing 5% skim milk, they were incubated with the primary antibodies overnight at 4°C. HRP-conjugated appropriate secondary antibodies were incubated at room temperature for 1 hour. A ChemiDoc Imaging System (Bio-Rad) was used to detect chemiluminescence.

The primary antibodies are as follows: anti-DDX3X antibody (Proteintech, Cat 11115-1-AP, RRID: AB_10896499), anti-AEP antibody (R&D Systems, Cat AF2199, RRID: AB_416565), anti-Flag antibody (Sigma-Aldrich, Cat F1804, RRID: AB_262044), anti-Ki-67 antibody (Abcam, Cat ab16667, RRID: AB_302459), anti- mCherry antibody (Abcam, Cat ab167453, RRID: AB_2571870), anti-β-tubulin antibody (Abcam, Cat#ab21058, RRID: AB_727045), anti-Lamin B1 antibody (Proteintech, Cat 12987-1-AP, RRID: AB_2136290), anti-U2AF2 antibody (Santa Cruz Biotechnology, Cat sc-53942, RRID: AB_831787), anti-SRSF1 antibody (Santa Cruz Biotechnology, Cat sc-33652, RRID: AB_628248), anti-hnRNPA1 antibody (Santa Cruz Biotechnology, Cat sc-32301, RRID: AB_627729), and anti-β-actin antibody (Abcam, Cat ab8226, RRID: AB_306371). The secondary antibodies were: HRP Rabbit Anti-Goat IgG (H+L) (Abclonal, Cat AS029, RRID: AB_2769859), HRP Goat Anti-Rabbit IgG (H+L) (Abclonal, Cat AS014, RRID: AB_2769854), HRP Goat Anti-Mouse IgG (H+L) (Abclonal, Cat AS003, RRID: AB_2769851).

### IP-MS analysis.

MEFs were isolated from AEP knockout C57BL/6J) mice. Then, *lgmn*-KO MEFs were infected with human *LGMN*-expressing lentivirus. Cells were lysed in IP buffer (Solarbio) and subjected to IP using an AEP antibody (dilution: 1:50, R&D Systems, Cat AF2199, RRID: AB_416565). A portion of the immunoprecipitate was applied for silver staining, and the rest was sent to Oebiotech Co, Ltd (Shanghai, China), for MS detection.

The MS detection of nuclear fractions of AEP KD/mCherry-tDDX3X rescue U87-MG cells was performed in a similar process. Cell fractionation was performed using Nuclear and Cytoplasmic Extraction Reagent (Cat# 78833, Thermo Fisher Scientific). Cell nuclear lysates were subjected to IP using an anti-mCherry antibody (dilution: 1:50, Abcam, Cat# ab167453, RRID: AB_2571870).

### RNA-IP analysis.

The RNA-IP (RIP) assay was performed using the Magna RIP RNA-binding protein IP kit (Sigma-Aldrich) according to the manufacturer’s instructions. Briefly, cells were harvested and lysed in RIP lysis buffer and then incubated with Dynabeads coated with Flag antibody (Sigma-Aldrich) or control IgG at 4°C overnight. The coprecipitated RNAs were reverse transcribed and analyzed by quantitative reverse-transcription PCR (qRT-PCR).

### Stable cell line preparation.

The lentivirus was packaged by cotransfection of the targeted shRNA construct or overexpression construct (Supplemental Table 11) with the helper plasmids pMD2.G and psPAX2 into Lenti-X 293T cells. The virus-containing supernatant was harvested and filtered through a 0.45 μm PVDF filter (VWR) and applied to the indicated cells in the presence of polybrene (5–10 μg/mL). After 72 hours, the cells were selected with puromycin.

### In vitro hypoxia and nutrient deprivation model.

Cells were cultured to a fusion level of approximately 80%, and the medium was replaced with EBSS (containing 200 μm/mL cobalt chloride) for the indicated times (0, 1, 2, or 4 hours).

### Cycloheximide chase protein stability assay.

For CHX treatment, HEK293T cells were grown under normal conditions to approximately 80% confluence in 6 cm plates. pHY-023-DDX3X, pHY-023-tDDX3X-N,and pHY-023-tDDX3X-C were then transfected into HEK293T cells, followed by 0, 4, 8, 12, 16,and 20 hours of treatment with 20 μg/mL cycloheximide after 24 hours of transfection. Cells were then harvested and lysed by scraping into RIPA lysis buffer with protease inhibitors (cocktail and PMSF). The protein concentration was determined with BCA assays for subsequent Western blotting analysis.

### Immunocytochemistry.

Cells, as indicated, were plated on round coverslips in 6-well plates and fixed with 4% polyformaldehyde at room temperature for 15 minutes before permeabilizing with 1% Triton X-100 for 2 minutes. Nuclei were stained with DAPI (Sigma-Aldrich) at room temperature for 1 minute. Images were captured by confocal microscopy (STELLARIS 5, Leica).

### CCK-8 assays and colony formation assays.

CCK-8 assays and colony formation assays were performed as described previously (17).

### Soft-gel colony assays.

A gel matrix with sterile low-melted agarose (Sangon Biotech) was prepared in a 24-well plate. The cells (approximately 2 × 10^4^) were mixed with the gel matrix to ensure an even distribution. The cell suspension was then plated onto a culture dish or plate, allowing the cells to settle within the gel. The gel matrix was allowed to solidify at 37°C. When the agarose developed a jelly-like consistency, a layer of complete culture medium was added to nourish the cells. Subsequently, the 24-well plate was placed in an incubator for cultivation. Every 48 hours, 500 μL of complete culture medium was added to ensure normal cell growth. The colonies were monitored periodically using microscopy or other imaging techniques. Colony size and number were assessed to evaluate the clonogenic potential and growth characteristics of the cells.

### Acquisition and culture of GBOs.

The strict procedure for obtaining GBOs from fresh GBM tissue samples can be found in the referenced literature (38).

### Molecular profiles of organoids and GBM tissues.

Molecular profiling was performed by Beijing Genomics Institute (BGI) Tech Solutions Co, Ltd, which provided sequencing services. Qualification-tested tumor tissues and GBOs were subjected to the following tests: IDH1 R132 mutant, IDH2 R172 mutant, H3F3A G35 mutant, H3F3A K28M mutant, HIST1H3B K28M mutant, HIST1H3C K28M mutant, TERT C228T and C250T, MGMT promoter methylation, BRAF V600E, EGFRvIII, KIAA1549-BRAF fusion, CNV Chr1p deletion, CNV Chr19q deletion, CNV Chr7 amplification, and CNV Chr10 deletion analyses.

### Lentivirus infection of GBOs.

The steps for lentiviral infection were slightly modified based on the referenced literature (38). We used a medium titer lentiviral solution and 10 μg/mL polybrene (Biosharp) as a transduction enhancer to coculture with GBO for 3 hours. Then, fresh GBO culture medium was replaced, and approximately 7 days later, significant fluorescence could be observed, indicating successful viral infection.

### In vivo xenograft model.

For the glioma model, athymic male nu/nu mice (Lingchang Biotech) were used. U87-MG cells (5 × 10^5^/5 μL) were stereotactically injected into the right ganglia region. Mice were monitored daily and examined by MRI scanning when they presented with weight loss or neurologic impairments. Tumor diameters were measured in the MRI images. The mice were euthanized when they were severely emaciated and depressed.

For the breast cancer model, athymic female nu/nu mice aged 6 weeks were used. MDA-MB-231 cells (1 × 10^6^) were inoculated into the left second mammary fat pads of the mice. After 40 days, the mice were sacrificed, and the tumors were isolated. Tumor volume was analyzed: volume = length × width^2^/2.

### Patient specimens.

This study included 95 glioma specimens and 6 breast cancer specimens that were clinically and histopathologically diagnosed at Ren Ji Hospital. Their diagnoses were independently rereviewed by 2 pathologists and classified by WHO criteria. Freshly frozen glioma tissues [LGG: *n* = 10, HGG: *n* = 72, recurrent glioma (RecG): *n* = 18] and 5 normal brain tissues were procured from the Neurosurgery Department of Ren Ji Hospital. Six sets of breast cancer and adjacent normal tissue pairs were acquired from the Breast Surgery Department of Ren Ji Hospital. Tumor tissues were rinsed with PBS to remove blood clots and tissue debris and then stored in an ultralow-temperature freezer.

### BiFC.

For BiFC analysis, we used yellow fluorescent protein (YFP). For analysis of the interaction between truncated DDX3X and SRSF1 and hnRNPA1, HEK293T cells were seeded in a 24-well plate at 100,000 cells/well in full medium 24 hours before transfection. The cells were transiently transfected with Lipofectamine 2000 (Thermo Fisher Scientific) following the manufacturer’s protocol in the following combinations: pHY-009-YC156-tDDX3X-C (truncated DDX3X fused to a C-terminal YFP fragment) with empty pHY-009-YN155 (an N-terminal YFP fragment only) or pHY-009-YC156 (a C-terminal YFP fragment only) with either pHY-009-YN155-SRSF1 or pHY-009-YN155-hnRNPA1 (an N-terminal YFP fragment fused to SRSF1 or hnRNAPA1, respectively) as a NC and pHY-009-YC156-tDDX3X-C with either pHY-009-YN155-SRSF1 or pHY-009-YN155-hnRNPA1. Cells were analyzed 48 hours after transfection using a laser scanning confocal microscope. All pictures were taken from regions with a comparable cell density.

### RNA-Seq and data analysis of AS events.

RNA was isolated from the stable AEP KD, AEP KD/tDDX3X-C res and NC groups of U87-MG cells (triplicate in each group) and subsequently subjected to paired-end RNA-Seq using the Illumina HiSeq 2000 system according to the manufacturer’s instructions. Read mapping and data analysis using rMATS software for differentially spliced exons/introns were carried out by OeBiotech (Shanghai, China). In the rMAT analysis, AS events are detected using junction counts. The selection process entails filtering based on 2 criteria across the 2 groups: an absolute difference in IncLevelDifference > 0.01 and an FDR ≤ 0.05. The pairwise comparison of 3 cellular subgroups revealed AS events coregulated by AEP and tDDX3X-C. Briefly, by intersecting the events with decreased frequency in the NC versus KD groups and the events with increased frequency in the KD versus tDDX3X-C rescue groups, we obtained the overlapping set of AS events, which demonstrated a positive correlation in coregulation by AEP and tDDX3X-C. Conversely, a negative correlation in coregulation was inferred when the opposite scenario occurred. RNA-Seq of alternative sites was performed by IGV software.

### Statistics.

Expression analysis and survival analysis of glioma and breast cancer were performed by GEPIA, a web server for tumor and normal gene expression profiling and interactive analyses (60). Correction analysis of 2 genes was also performed with GEPIA.

Statistical analyses were performed using SPSS 21.0 software. Statistical graphs were drawn using GraphPad Prism 9 software (GraphPad Software, Inc.). 2-tailed Student’s *t* tests, 1-way ANOVAs, Pearson correlation analyses, Kaplan-Meier analyses, and log-rank tests were used to analyze the corresponding data. A 2-tailed *P* value less than 0.05 was considered significant.

### Study approval.

Ethics approval (IRB number, RA-2022-031) and prior patient consent were obtained from the ethics committee of Ren Ji Hospital, School of Medicine, Shanghai Jiao Tong University. Mice were operated on and housed according to the criteria outlined in the Guide for the Care and Use of Laboratory Animals. Animal experimental procedures were approved by the Institutional Animal Care and Use Committee of Shanghai Jiao Tong University, School of Medicine (IRB number, B-2022-091).

### Data availability.

RNA-Seq data of the AEP KD, AEP KD/tDDX3X-C res and NC groups of U87-MG cells have been deposited in the Sequence Read Archive (SRA) [https://submit.ncbi.nlm.nih.gov/subs/sra/] under the accession code PRJNA911018. Single nuclear sequences of GBM tissues have been deposited in the Genome Sequence Archive in the National Genomics Data Center, China National Center for Bioinformation/Beijing Institute of Genomics, Chinese Academy of Sciences (GSA-Human: HRA003631), which are publicly accessible at [https://ngdc.cncb.ac.cn/gsa-human] All the other data supporting the findings of this study are available within the article and its supplementary information files. Values for all data points in graphs are reported in the Supporting Data Values file.

## Author contributions

YL and JC conceptualized the study. YL and KL designed the study methodology. WZ, LC, JY, SZ, JZ, ZS, KL, BC, and HL conducted the investigations. Haiwei Wang performed the bioinformatics analysis. WZ and LC wrote the original draft of the manuscript. YL, JG, JZ, ZQ, HX, LW, Hongxiang Wang, CM, and YQ reviewed and edited the manuscript. YL, KL, SZ and YQ acquired funding for the study. YL, JC, JG, YQ, HL, and SZ acquired resources for the study. YL acted as project administrator. YL and JC supervised the study. The order of co–first authors was determined by the volume of work each contributed to the study.

## Supplementary Material

Supplemental data

Supplemental tables 1-11

Supporting data values

## Figures and Tables

**Table 1:**
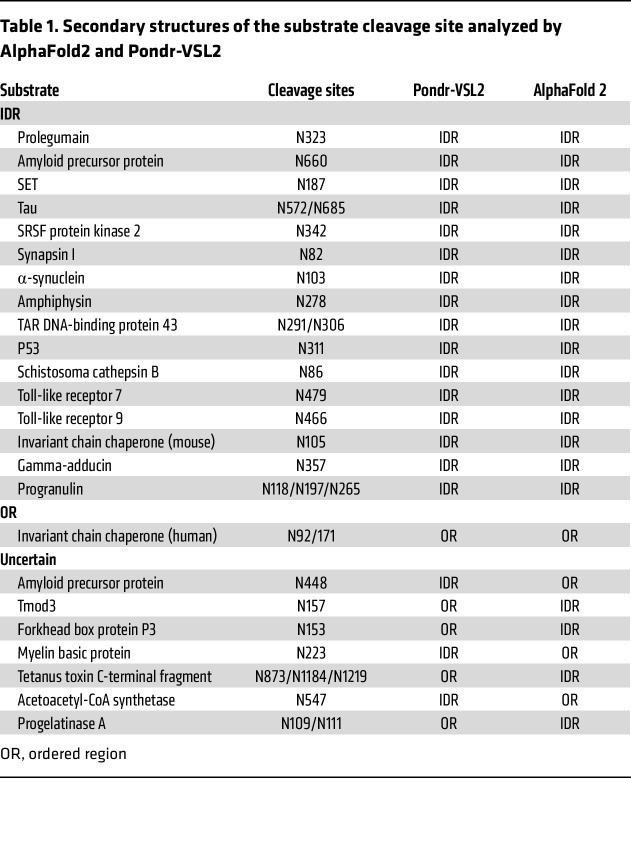
Secondary structures of the substrate cleavage site analyzed by AlphaFold2 and Pondr-VSL2

## Version 1 11/21/2023

In-Press Preview

## Version 2 02/01/2024

Electronic publication
