# Supplementary material for: AEP-cleaved DDX3X induces alternative RNA splicing events to mediate cancer cell adaptation in harsh microenvironments
Source: J Clin Invest. 2024 Feb 1;134(3):e173299. doi: 10.1172/JCI173299 (PMC10849765; doi:10.1172/JCI173299)
Supplement: Supplemental data [file jci-134-173299-s131.pdf]

Supplemental Material

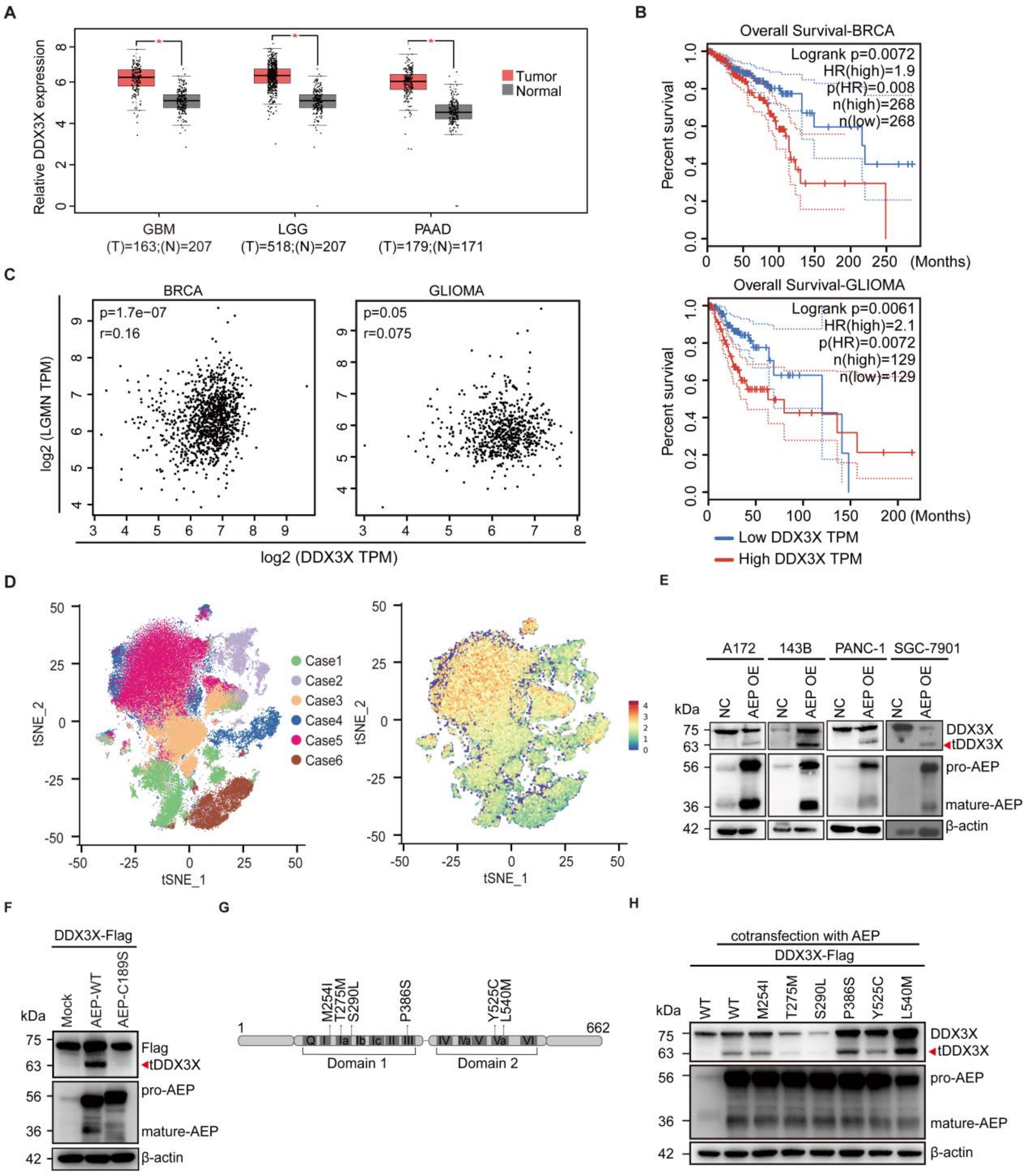

Additional file 1: Supplemental Figure 1. Expression and prognostic significance of DDX3X in many types of tumours.

(A) The transcript levels of DDX3X in GBM, LGG and PAAD tissues were analysed via GEPIA based on the TCGA database. GEPIA is an online website for bioinformatics analysis (http://gepia.cancer-pku.cn/). **(B)** Survival analysis in BRCA and glioma (including LGG and GBM) patients based on DDX3X expression. **(C)**

Correlation analysis of AEP and DDX3X based on TCGA expression data. (**D)** Expression of DDX3X in GBM were analysed by single nuclear RNA-seq (snRNA-seq) of 6 cases of GBM. **(E)** Immunoblots of DDX3X, AEP and β-actin expression in A172, PANC-1, 143B and SGC-7901 cells with or without AEP overexpression (AEP OE). **(F)** Immunoblots of Flag-tagged DDX3X, AEP and β-actin in HEK293T cells cotransfected with DDX3X and wild-type AEP (enzyme active type) or C189S mutant-AEP (enzyme inactive type). **(G)** Natural mutation site is indicated on the structure diagram of DDX3X. **(H)** Immunoblots of Flag-tagged DDX3X, AEP and β-actin expression in HEK293T cells cotransfected with wild-type or mutant flag-tagged DDX3X and AEP.

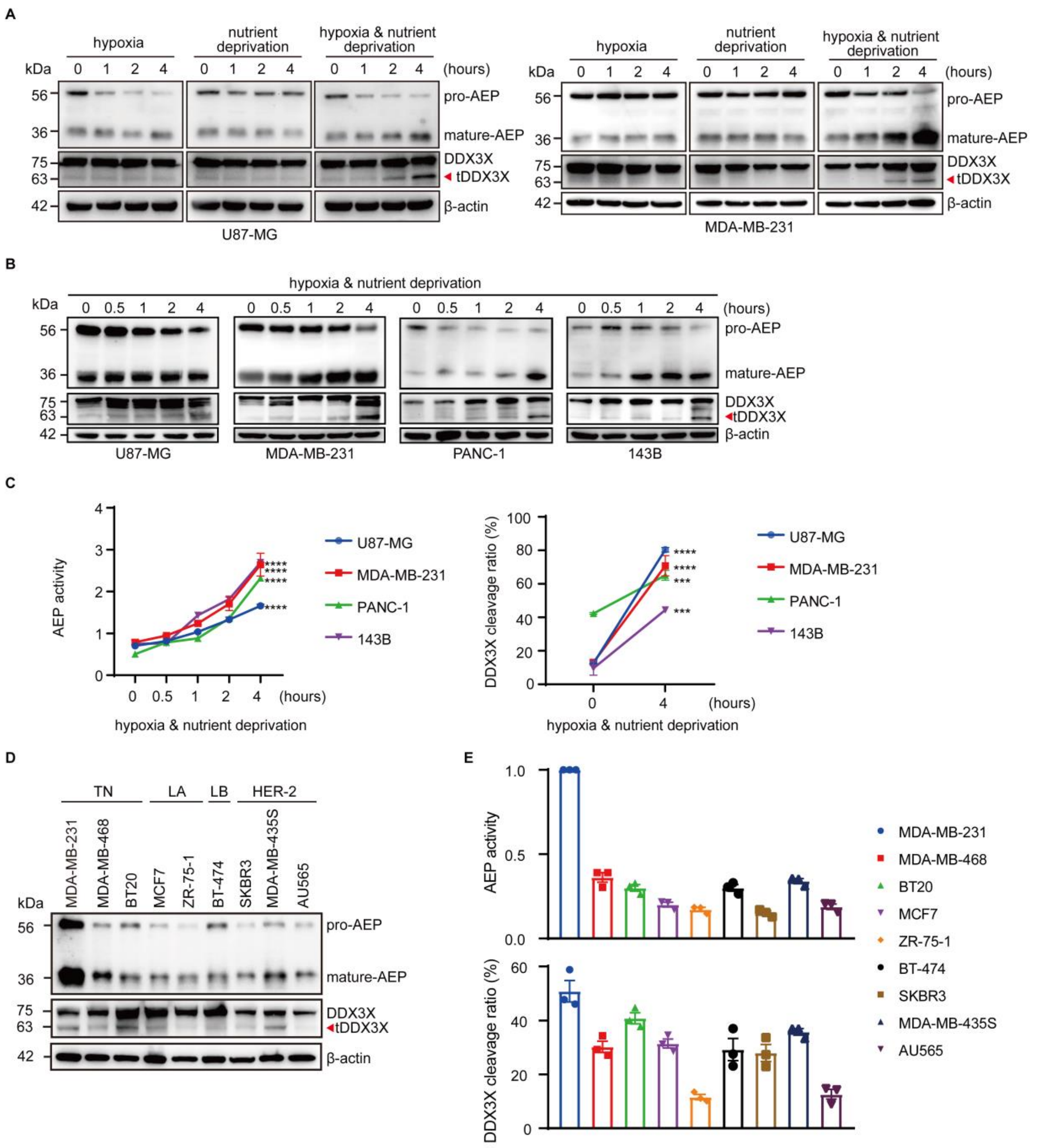

Additional file 2 Supplemental Figure 2. Under stress conditions, AEP cleaves DDX3X in multiple cancer cells in a HIF1A-dependent manner.

(A) Immunoblots of DDX3X, AEP and β-actin expression in cancer cells (U87-MG, MDA-MB-231) under only hypoxia, only nutrients deprivation, and combination stimulus of hypoxia and nutrients deprivation, respectively. **(B)** Immunoblots of DDX3X, AEP and β-actin expression in cancer cells (U87-MG, MDA-MB-231, PANC-1 and 143B) under the hypoxia and nutrients deprivation. **(C)** Quantitation of

the AEP activity (mature-AEP/pro-AEP) and DDX3X cleavage ratio (tDDX3X/DDX3X) in the cells mentioned in (B). **(D)** Immunoblots of DDX3X, AEP and β-actin expression in four different subtype of breast cancer cells. **(E)** Relative quantitation of the AEP activity (mature-AEP/pro-AEP) and DDX3X cleavage ratio (tDDX3X/DDX3X) in the cells mentioned in (D). Data were plotted as the mean ± SEM. Statistical analysis was performed using one-way ANOVA followed by Dunnett's multiple comparisons test (C). ***P < 0.001, ****P < 0.0001. Data shown are representative of 3 independent experiments.

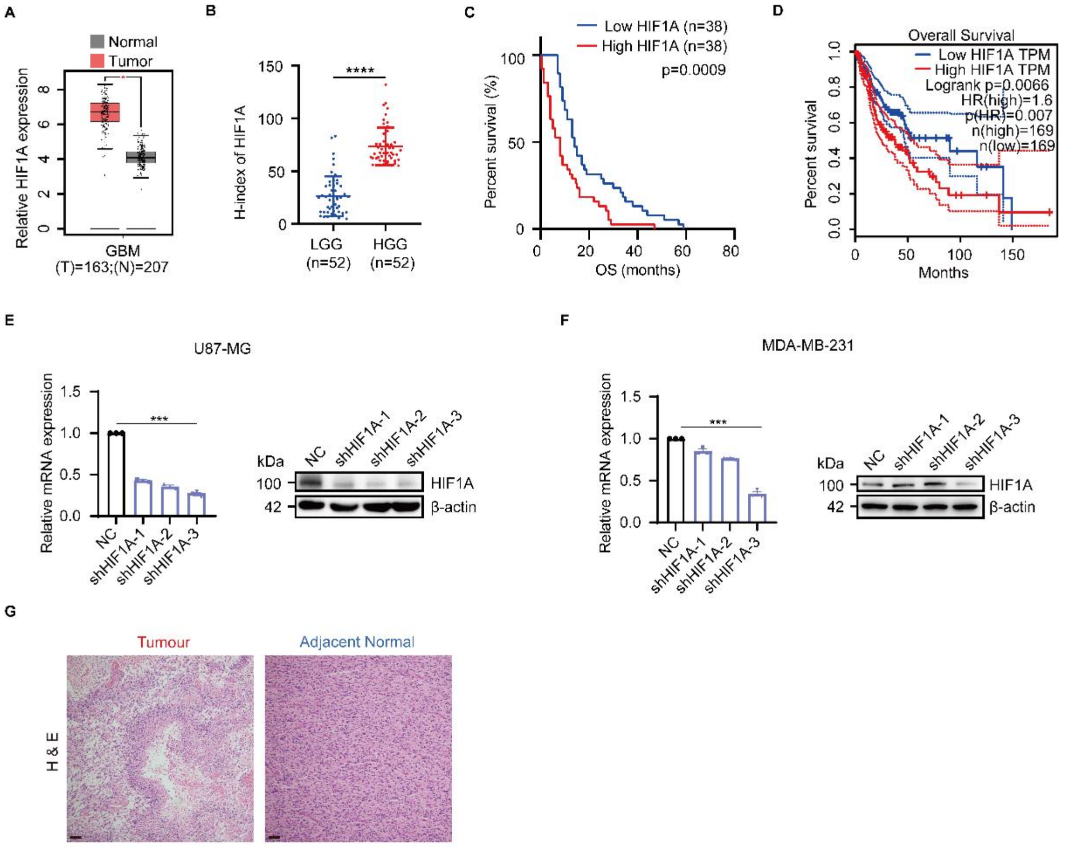

Additional file 3 Supplemental Figure 3. High HIF1A associated with poor prognosis of glioma.

(A) The transcript levels of *HIF1A* in GBM tissues were analysed by GEPIA. **(B)** Scatter plots of *HIF1A* expression in high-grade glioma (HGG) and low-grade glioma

(LGG). **(C)** Kaplan-Meier survival curve of GBM patients expressing high (H-index >40) or low (H-index <38) HIF1A from the glioma TMA cohort. **(D)** Survival analysis of *HIF1A* by GEPIA based on the TCGA database. **(E, F)** RT-qPCR was used to detect the relative mRNA expression of HIF1A after HIF1A knockdown in cancer cells (U87-MG and MDA-MB-231) and corresponding immunoblots of HIF1A were shown. (**G**) Representative images of H & E staining from tumour and adjacent normal parts of GBM. Scale bar: 20 μm. Data were plotted as the mean ± SEM. Statistical analysis was performed using one-way ANOVA followed by Dunnett's multiple comparisons test (E, F) and unpaired t test (B). ***P < 0.001, ****P < 0.0001. Data shown are representative of 3 independent experiments.

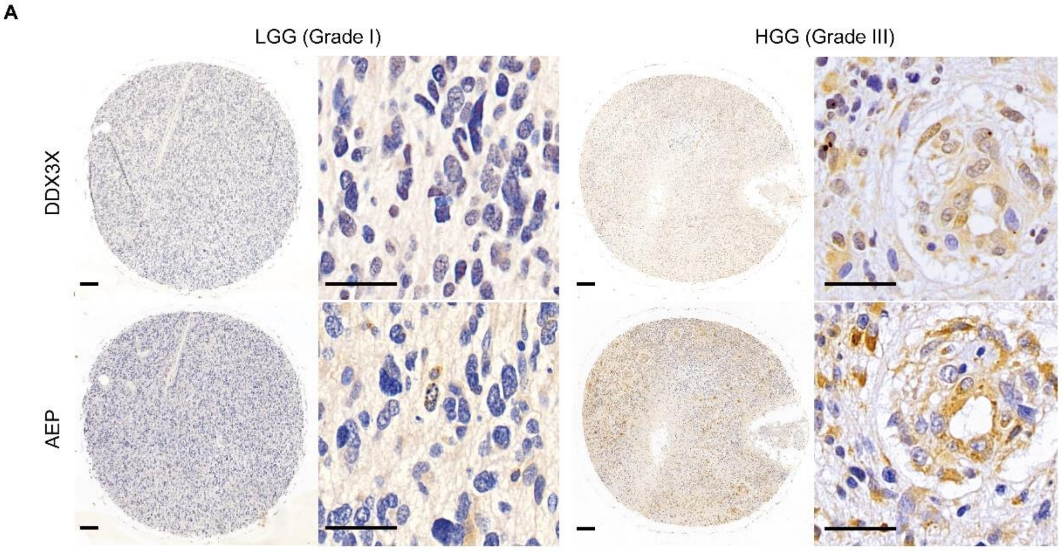

Additional file 4: Supplemental Figure4. Expression of AEP and DDX3X in glioma tissue microarrays.

(A) Representative images of IHC staining of AEP and DDX3X in glioma tissue-microarrays containing different WHO grades (grade I and III). Scale bars: 200 μm for overview; 50 μm for enlargement.

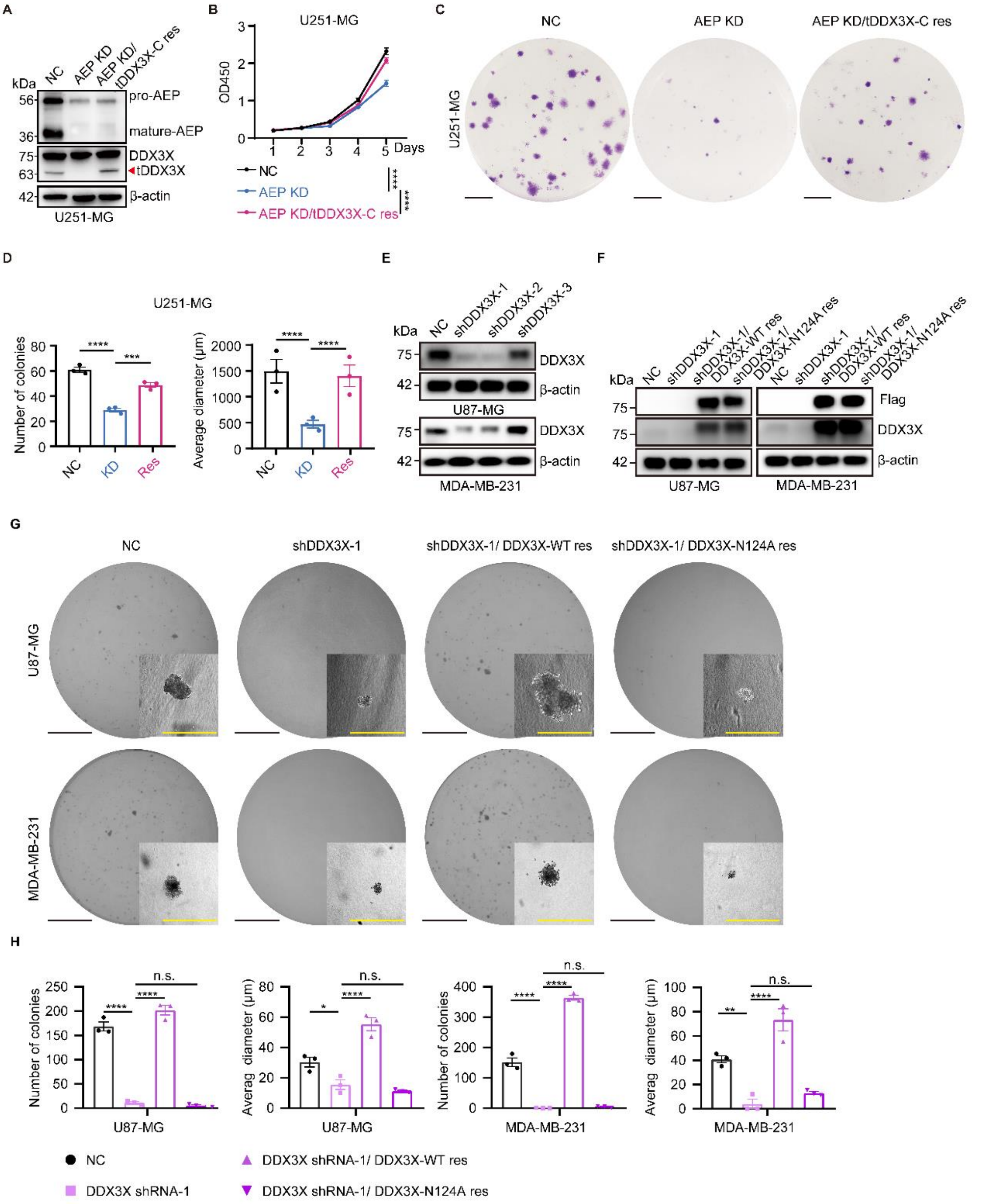

Additional file 5: Supplemental Figure 5. tDDX3X-C produced by AEP promotes tumour progression in vitro and in vivo.

(A) Immunoblots of AEP, DDX3X and β-actin expression in U251-MG cells in the following groups: negative control (NC), AEP knockdown (AEP KD) or AEP knockdown & tDDX3X-C rescue (AEP KD/tDDX3X-C res). **(B)** CCK-8 assays of

U251-MGcells in the following groups: NC, AEP KD, AEP KD/tDDX3X-C res. **(C)** Colony assays of U251-MG cells in the following groups: NC, AEP KD, AEP KD/tDDX3X-C res. Scale bars: 6 mm for overview. (**D**) The number and average diameter (μm) of the colony were measured by Image J. **(E)** Immunoblots of DDX3X and β-actin in U87-MG and MDA-MB-231 cells with or without DDX3X knockdown. **(F)** Immunoblots of Flag, DDX3X and β-actin expression in U87-MG and MDA-MB-231 in the following groups: negative control (NC), DDX3X knockdown (shDDX3X-1), DDX3X knockdown and DDX3X wild-type rescue (shDDX3X-1/DDX3X-WT res) or DDX3X knockdown and DDX3X N124A rescue (shDDX3X-1/DDX3X-N124A res). **(G)** Representative soft agar colony formation assays of U87-MG and MDA-MB-231 cells in the following groups: NC, shDDX3X-1, shDDX3X-1/DDX3X-WT res, shDDX3X-1/DDX3X-N124A res. Scale bars: 6 mm for overview; 100 μm for enlargement. **(H)** Histogram of number of colonies and average diameter of colonies of U87-MG and MDA-MB-231 mentioned in **(G).** Data were plotted as the mean ± SEM. Statistical analysis was performed using one-way ANOVA followed by Tukey's (B) or Sidak's multiple comparisons test (D, H). *P < 0.05, **P < 0.01, ***P < 0.001, ****P < 0.0001. Data shown are representative of 3 independent experiments.

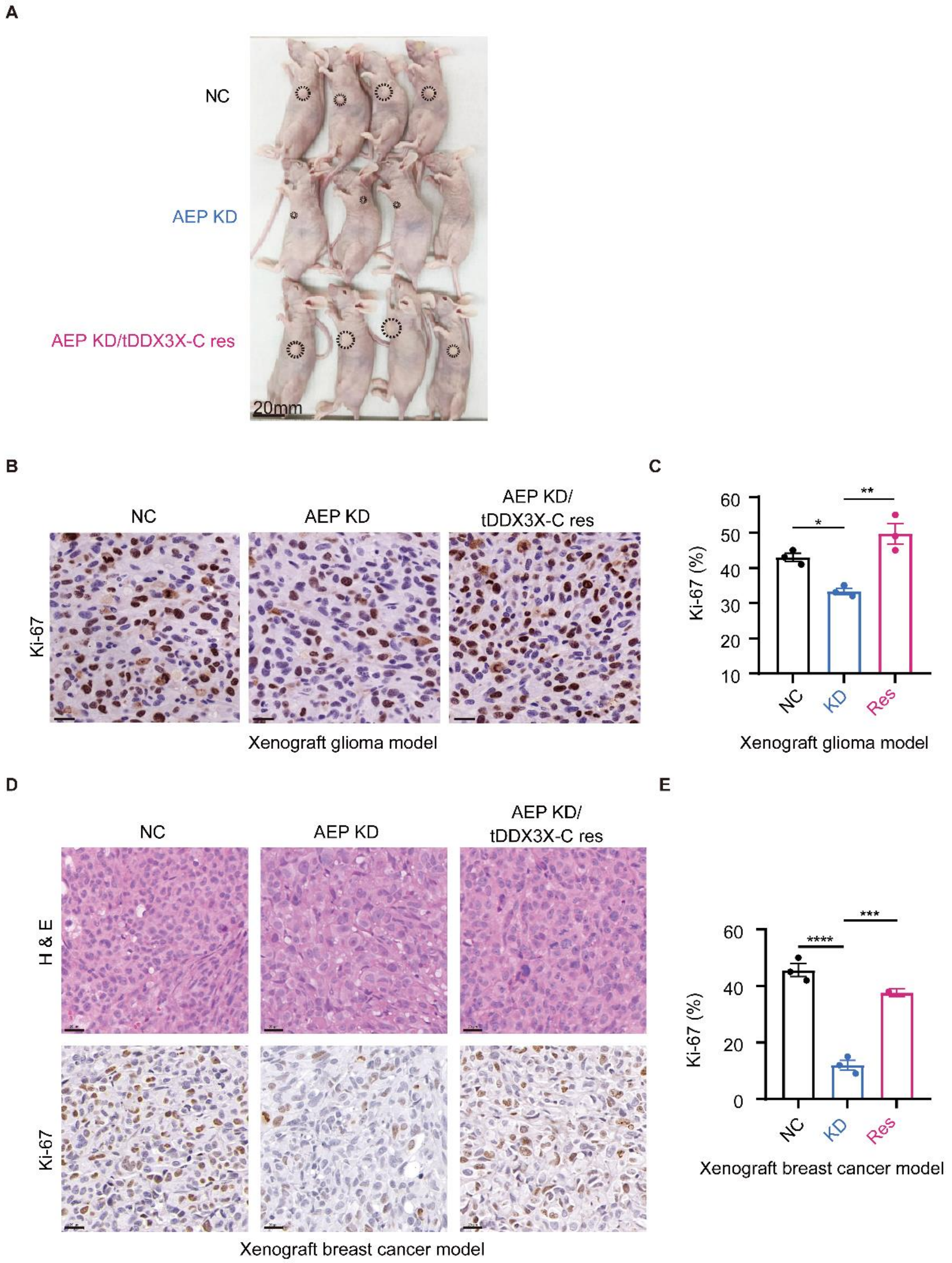

Additional file 6: Supplemental Figure 6. tDDX3X-C produced by AEP promotes tumour proliferation.

(A) Xenograft breast cancer models of MDA-MB-231 cells in nude mice grouped by NC, AEP KD, and AEP KD/tDDX3X-C res. Scale bar: 20 mm. **(B)** Ki-67 expression was detected in tumours from mice inoculated with NC, AEP KD, AEP

KD/tDDX3X-C res U87-MG cells to analyse the proportion of proliferating cells. Representative IHC images are shown. Scale bar = 40 μm. **(C)** The ratio of proliferative cells in (B) was determined using ImageJ. (**D)** Representative H & E images of the xenograft breast cancer model grouped by NC, AEP KD, AEP KD/tDDX3X-C res; Ki-67 expression was detected and analysed in the same way. Scale bar = 20 μm. **(E)** The ratio of proliferative cells in (D) was determined using ImageJ. Data were plotted as the mean ± SEM. Statistical analysis was performed using one-way ANOVA followed by Sidak's multiple comparisons test (C, E). $^{*}P < 0.05$, $^{**}P < 0.01$, $^{***}P < 0.001$, $^{****}P < 0.0001$. Data shown are representative of 3 independent experiments.

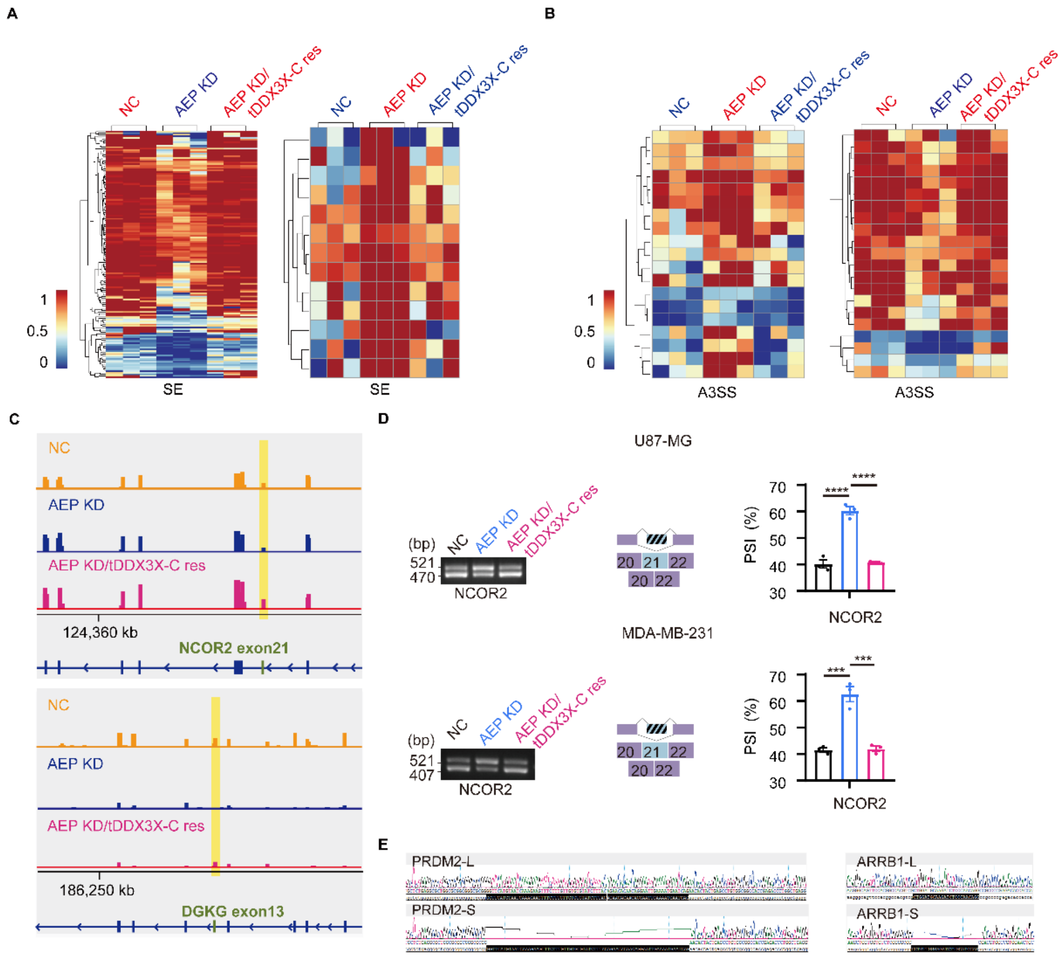

Additional file 7: Supplemental Figure 7. AEP promotes AS of widespread pre-mRNAs via tDDX3X-C.

(A, B) Representative heatmap for changed AS events from **(A)** skipped exons (SEs) and **(B)** alternative 3' splice sites (A3SSs) positively or negatively regulated by AEP /tDDX3X-C. **(C)** RNA-seq results of alternative sites in *DGKD* and *NCOR2* using IGV software analysis. **(D)** PCR and AGE (agarose gel electrophoresis) analyses for *NCOR2* exon 21 regulated by AEP /tDDX3X-C in U87-MG and MDA-MB-231 cells. The middle panels represent the schematic diagram of the indicated AS exons. Right panels show the quantification of percent spliced in (PSI). (**E**) Sanger sequencing of

different isoform PCR products. L indicates the long isoform in which the exon has spliced; in contrast, S indicates the short isoform in which the exon has skipped. Data were plotted as the mean ± SEM. Statistical analysis was performed using one-way ANOVA followed by Tukey's multiple comparisons test (D). ***P < 0.001, ****P < 0.0001. Data shown are representative of 3 independent experiments.

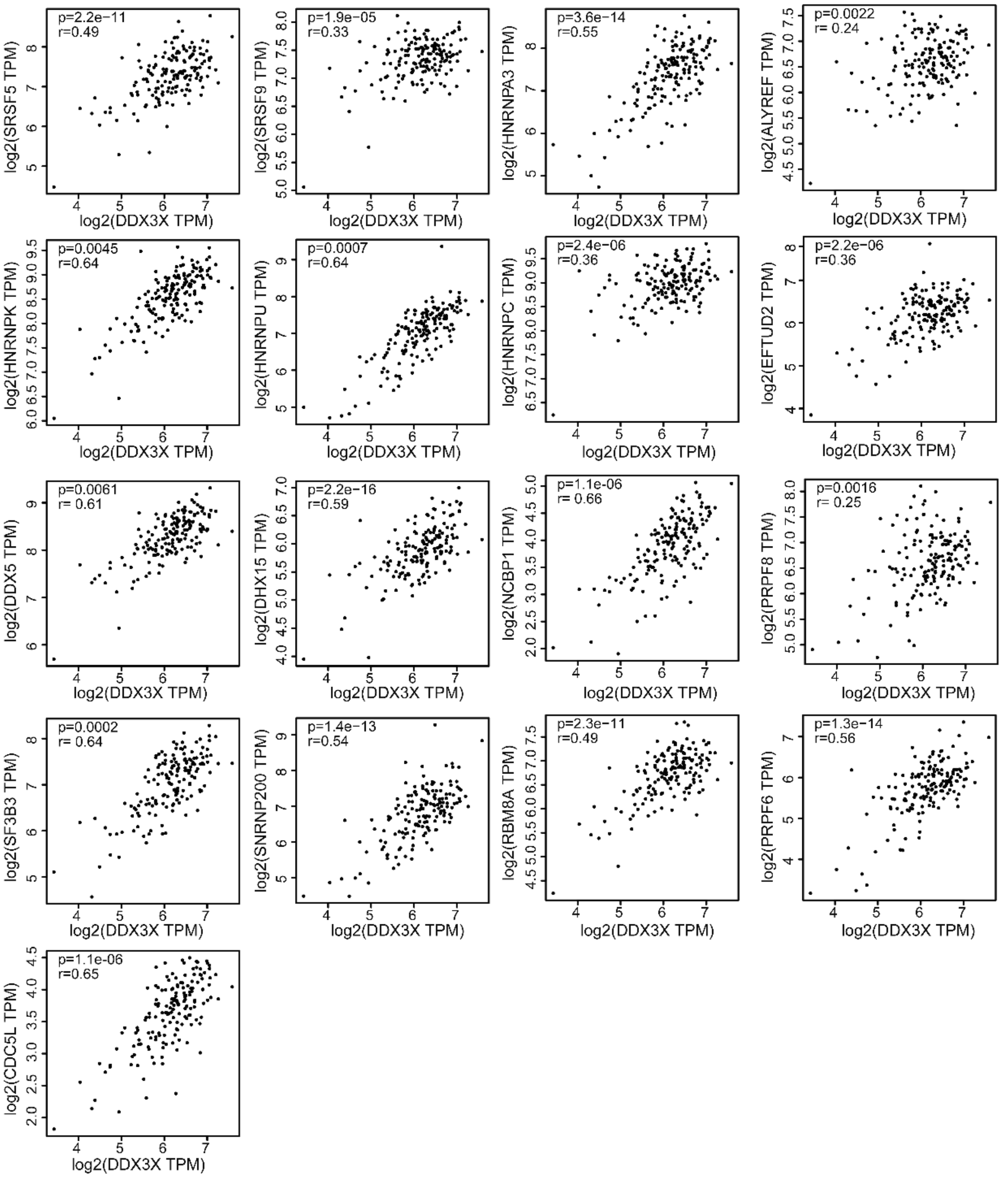

Additional file 8: Supplemental Figure 8. Expression correction analysis of DDX3X and splicing factors.

Expression correction analysis of DDX3X and splicing factors, including SRSF5, SRSF9, HNRNPA3, ALYRE, HNRNPK, HNRNPU, HNRNPC, EFTUD2, DDX5, DHX15, NCBP1, PRPF8, SF3B3, SNPNP200, RBM8A, PRPF6 and CDC5L.

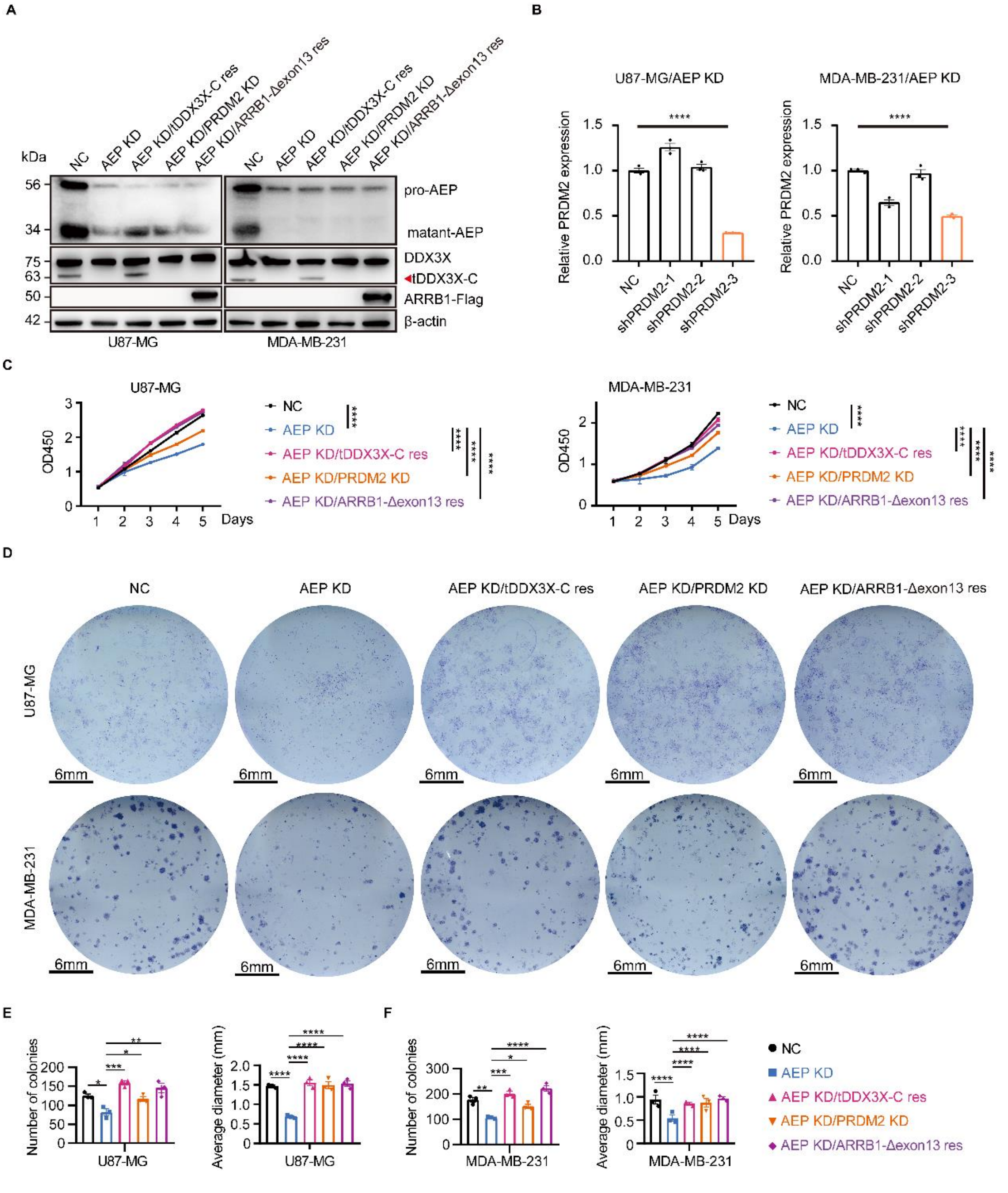

Additional file 9: Supplemental Figure 9. Alternative splicing of *PRDM2* and *ARRB1* are important for AEP/tDDX3X-C-mediated tumour growth.

(A) Immunoblots of AEP, DDX3X, ARRB1 and β-actin in U87-MG and MDA-MB-231 cells in the following groups: the negative control (NC), AEP knockdown (AEP KD), AEP knockdown & tDDX3X-C rescue (AEP KD/tDDX3X-C res), AEP knockdown & PRDM2 knockdown (AEP KD/PRDM2 KD) and AEP knockdown & ARRB1-△exon13 rescue (AEP KD/ARRB1-△exon13 res). **(B)** The relative mRNA expression of PRDM2 after PRDM2 knockdown was detected by qPCR. **(C)** The CCK-8 assays of cells constructed in (A). **(D)** Representative plate colony assays of cells constructed in (A). Scale bar: 6 mm. **(E-F)** The number and average diameter (μm) of colonies were determined by ImageJ. Data were plotted as the mean ± SEM. Statistical analysis was performed using one-way ANOVA followed by Dunnett's multiple comparisons test (B), Tukey's multiple comparisons test (C) and Sidak's multiple comparisons test (E, F). *P < 0.5, **P < 0.01, ***P < 0.001, ****P < 0.0001. Data shown are representative of 3 independent experiments.

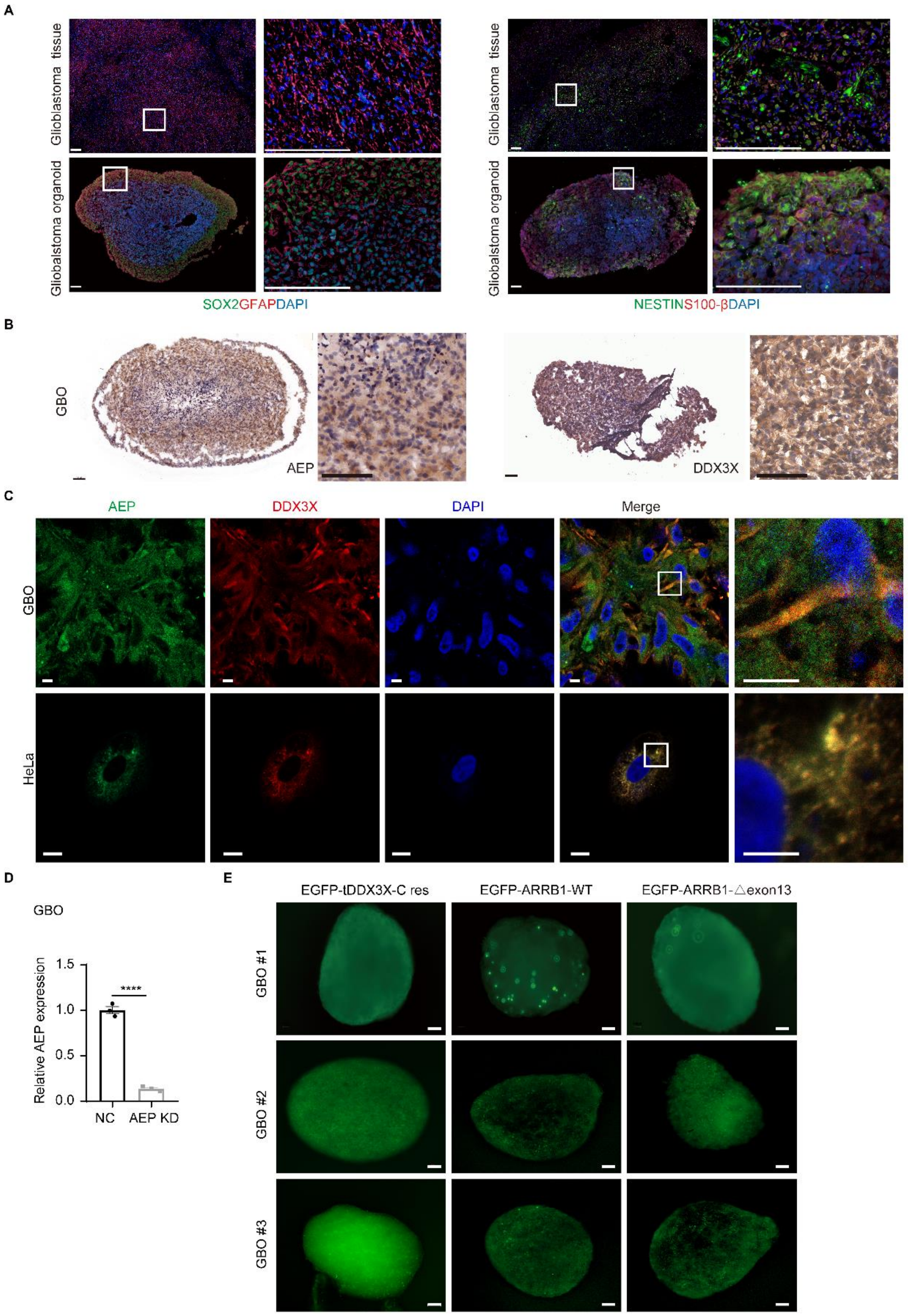

Additional file 10: Supplemental Figure 10. Interaction of AEP and DDX3X in GBOs.

(A) Immunofluorescence was used to detect glioma cells marker SOX2, GFAP

NESTIN, S100-β in tumour tissues and homologous GBOs. Scale bar: 200 μm. **(B)** Detection of AEP and DDX3X in GBOs by IHC. Scale bar: 100 μm. **(C)** Co-location assay of AEP and DDX3X in freeze section of GBO or HeLa cells by immunofluorescence. Scale bar: 10 μm. **(D)** GBOs were collected to detect AEP mRNA expression 14 days after lentivirus infection of glioma organoids. **(E)** Lentiviral infection system was used to express the corresponding genes in GBOs after AEP knockdown. Scale bar: 100 μm. Data were plotted as the mean ± SEM. Statistical analysis was performed using unpaired t test (D). ****P < 0.0001. Data shown are representative of 3 independent experiments.

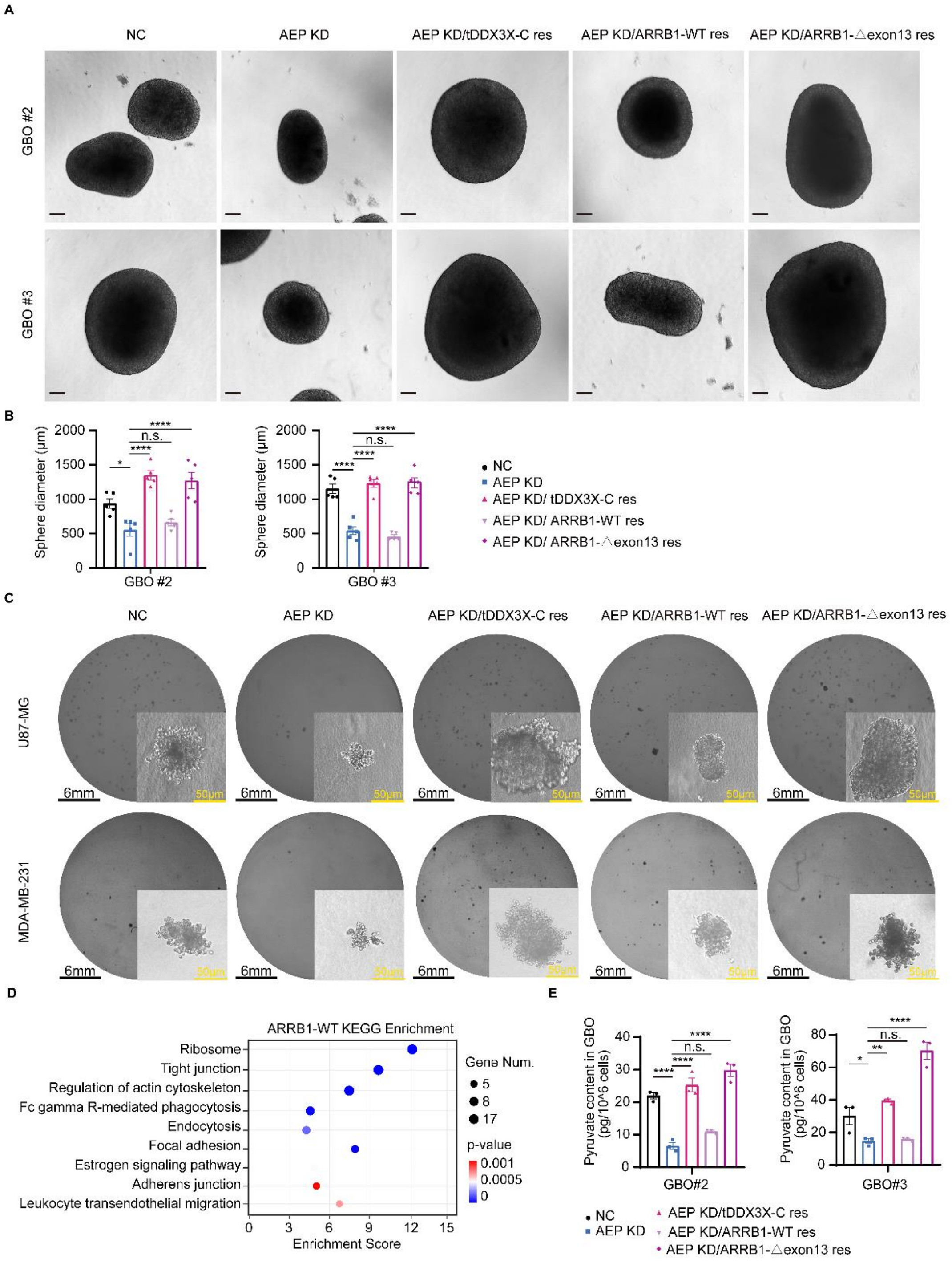

Additional file 11: Supplemental Figure 11. ARRB1-△Exon13 proteoform is critical for AEP/tDDX3X-C-mediated sustained tumour growth.

(A) Representative bright field images of GBO#2 and GBO#3 were used to show cell viability of GBOs. Scale bar: 200 μm. **(B)** Histogram of the diameter of GBO#2 and GBO#3, n = 5. **(C)** Representative soft agar colony formation assays of U87-MG

and MDA-MB-231 cells in the NC, AEP KD, AEP KD/tDDX3X-C res, AEP KD/ARRB1-WT res, and AEP KD/ARRB1-△exon 13 res groups. Scale bars: 6 mm for overview; 50 μm for enlargement. **(D)** Protein binding profile of ARRB1-WT detected by IP-MS. **(E)** Pyruvate content in the corresponding groups of GBO#2 and GBO#3. n = 5. Data were plotted as the mean ± SEM. Statistical analysis was performed using one-way ANOVA followed by Sidak's multiple comparisons test (B, E). *P < 0.05, **P < 0.01, ****P < 0.0001. Data shown are representative of 3 independent experiments.

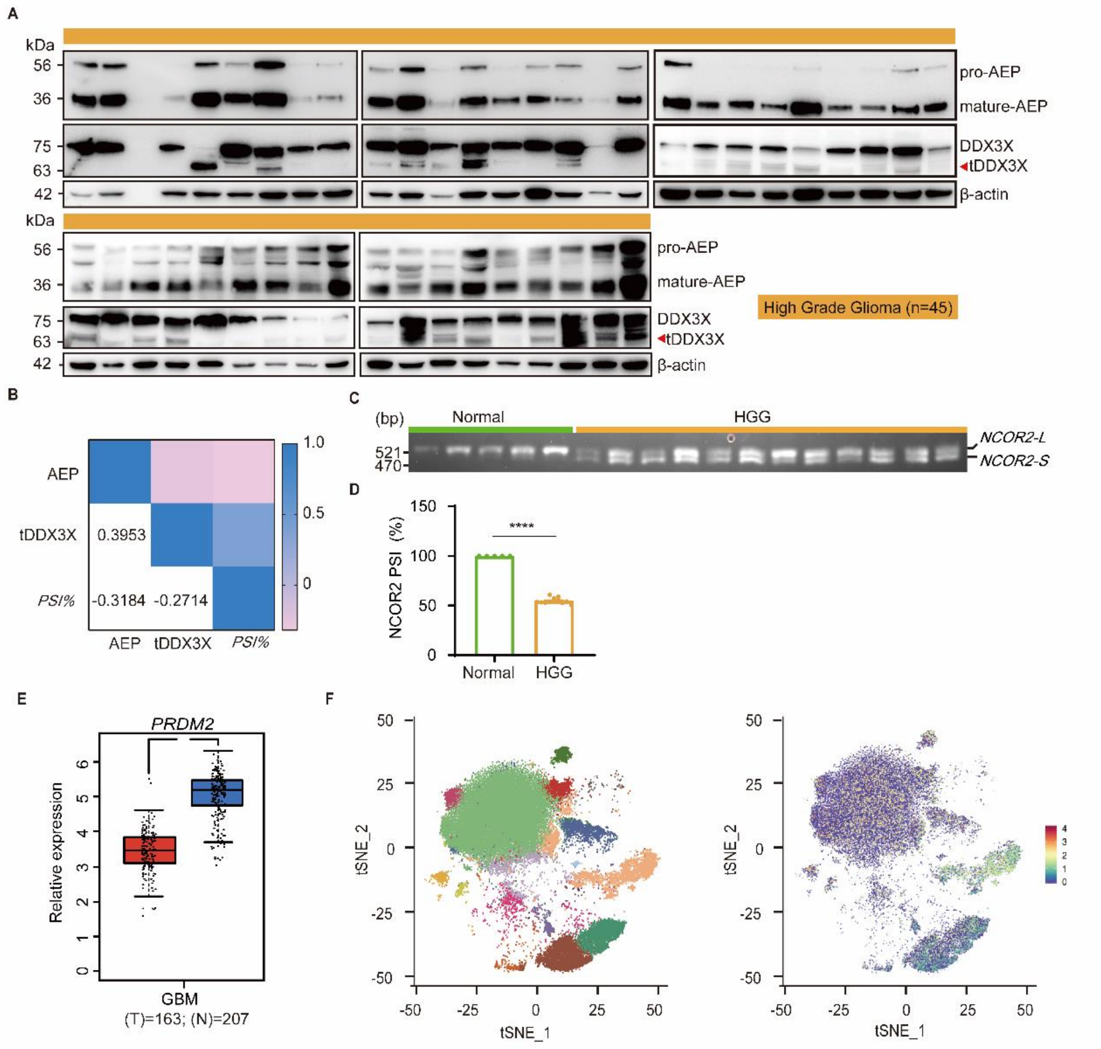

Additional file 12: Supplemental Figure 12. Alternative splicing is highly prevalent in cancerous tissues.

(A) Immunoblots of AEP, DDX3X and β-actin in high grade glioma cancerous tissues, HGG (n = 45). **(B)** Multiple correlation analysis of AEP expression, DDX3X cleavage ratio, and PSI for *ARRB1-△exon13*. **(C)** PCR and AGE analyses for *NCOR2△ exon21* in normal brain tissues (n = 5) and high-grade glioma (n = 12). **(D)** Quantification of the PSI based on PCR-AGE results of (C). **(E)** The transcript levels of PRDM2 in GBM tissues were analysed by GEPIA. **(F)** Single-nuclear RNA-seq (snRNA-seq) of 6 cases GBM showed that PRDM2 is lowly expressed in majority GBM cells at the transcriptome level. The Spearman correlation test was considered to indicate statistical significance in (B). Statistical analysis was performed using one-way ANOVA followed by unpaired t test (D, E) ****P < 0.0001. A heatmap was drawn by the online website Heatmapper (www.heatmapper.ca).

Additional file 13: Supplemental Table 1. Hif1a H-index of overall survival in glioma samples.

Additional file 14: Supplemental Table 2. Expression of hif1a in glioma samples of different grades.

Additional file 15: Supplemental Table 3.The known substrates of AEP

Additional file 16: Supplemental Table 4. Nuclear DDX3X rate in glioma samples of different grades.

Additional file 17: Supplemental Table 5. Correlation of AEP expression and the nuclear DDX3X staining rate in glioma samples.

Additional file 18: Supplemental Table 6. Differentially expressed genes regulated by AEP and tDDX3X.

Additional file 19: Supplemental Table 7. Alternative splicing events regulated by AEP and tDDX3X.

Additional file 20: Supplemental Table 8. MS analysis of IP tDDX3X-C.

Additional file 21: Supplemental Table 9. Molecular profiling of GBM tissues and GBOs.

Additional file 22: Supplemental Table 10. MS analysis of IP ARRB1 WT or delta exon 13.

Additional file 23: Supplemental Table 11. Primers and shRNA.

Supplementary method

Silver staining

For silver staining, the Quick Silk Staining Kit (Beyotime Biotechnology, Shanghai, China) was used. The gels were fixed in stationary liquid for more than 20 min, washed with 30% ethyl alcohol, washed again with Milli-Q grade pure water or double distilled water, sensitized, washed twice with water, and subsequently stained with silver solution (1×) for 10 min at room temperature. Next, the cells were washed with ddH_2O for 1 min, silver dye solution was added, and the cells were shaken on a shaker at room temperature for 3-10 min until the desired protein band appeared. Eventually, the reaction was stopped with stop solution (1 ×), and images were captured.

Plasmid construction and transfection

DDX3X was PCR amplified from cDNA obtained from HEK293T cells. Flag-tagged DDX3X and its point mutants tDDX3X-N, tDDX3X-C, U2AF2, SRSF1, and HNRNPA1 were cloned and inserted into the pHY-023 vector (Hanyin, Shanghai, China). mCherry-fused DDX3X-FL, tDDX3X-C, NES-tDDX3X-C, DDX3X mut-NES, DDX3X ΔNES, and tDDX3X-C mutNLS1&2 were cloned and inserted into the pHY-009 vector (Hanyin. Shanghai, China). Primers are listed in **Supplementary Table 1**. All constructs were extracted from transformed DH5α bacteria via an Endotoxin Free Plasmid Extraction Kit (Cat# DP108, Tiangen Biotech, Beijing) and verified by Sanger sequencing (Genewiz, Suzhou, China). For transient

expression of related proteins in HEK293T cells, Lipofectamine 2000 (Cat# 11668019, Thermo Fisher Scientific, USA) was used as described previously (1).

Haematoxylin-eosin (H&E) staining

For H&E staining, paraffin-embedded sections were dyed with haematoxylin and eosin dye as indicated (1). After dehydration, neutral resin was used for sealing, and images were captured by a Pannoramic Scanner (Pannoramic DESK, P-MIDI, 3D HISTECH, Hungary).

IHC

The expression of AEP and DDX3X was examined by IHC using a human glioma tissue microarray (T10-021, T14-431 and T011-298) containing 309 glioma samples from the Department of Neurosurgery, Ren Ji Hospital, School of Medicine, Shanghai Jiao Tong University; among them, LGG (n = 59), HGG (n = 199), and the remaining tumour tissue had only diagnostic information that lacked WTO grade information; IHC staining was performed according to a previously described protocol (1). Anti-AEP (1:50) antibodies and anti-DDX3X (1:200) were used. Images were captured by a Pannoramic Scanner (Pannoramic DESK, P-MIDI, 3D HISTECH, Hungary). Aipathwell (Solarbio, Wuhan), a digital pathological image analysis software based on artificial intelligence learning, was used to analyse each item on the tissue microarray. We used the H-index for semiquantification of the staining intensity of the samples. The H-score formula was as follows: H-score $=\sum (pi\times i)$ = (percentage of weakly stained cells × 1) + (percentage of moderately stained cells × 2) + (percentage of strongly stained cells × 3). The percentage of positive cells at a given

intensity (pi) was grouped as follows: 0–5%, 6–25%, 26–50% and more than 50%; i indicates the staining intensity, which was classified as follows: 0 (no staining), 1 (weak staining), 2 (moderate staining) and 3 (strong staining). The H-score is a value between 0 and 300; the higher the value is, the stronger the combined positive intensity is. These scores were determined by two independent pathologists in a blinded manner.

Immunofluorescence (IF)

Tissue slides were retrieved from the freezer and placed on a hot plate set at 55°C for 30 min to allow drying. Once the slides reached room temperature, they were inserted into holders and washed in a staining dish using 1x TBST buffer to remove OCT.

Three washes of the tissue slides were performed with 1x TBST buffer, and each wash lasted 5 min. Excess water was removed from the slides at room temperature using a water absorption method. The tissue sections were removed using a hydrophobic barrier pen and allowed to air dry for 5 min. Each tissue section was incubated at room temperature with approximately 100 μl of blocking and permeation buffer for 1 hour. The blocking and permeation buffer was removed, and the primary antibody, diluted in antibody dilution buffer (~100 μl, dilution: 1:50), was added to the tissue sections. SOX2 (CST, Cat#4900s), NESTIN (CST, Cat#33475), GFAP (CST, Cat#12389s), S100-β (Abcam, Cat#ab52642), and Ki-67 (Abcam, Cat# ab16667) were used. The samples were incubated overnight at 4°C. On the following day, the primary antibody solution was removed, and the tissue slides were quickly washed three times in the staining dish using 1x TBST buffer, with each wash lasting 5 min.

The tissue sections were incubated at room temperature with approximately 100 μl of secondary antibody (dilution: 1:500) and DAPI (dilution: 1:10000), both diluted in antibody dilution buffer, for 1.5 hours. The secondary antibody and DAPI were typically diluted at a ratio of 1:500. The secondary antibody solution was removed, and three quick washes of the tissue slides in the staining dish were performed using 1x TBST buffer, with each wash lasting 5 min. The tissue slides were washed once with 1x TBS buffer for 5 min to remove any residual detergent. The tissue sections were incubated with 1x autofluorescence quenching solution for 1 min. The autofluorescence quenching solution was rinsed off, and the tissue slides were washed three times using 1x TBS buffer, with each wash lasting 5 min. The slides were mounted by applying approximately 50-100 μl of mounting medium to each slide and covering them with a coverslip. The slides were allowed to dry overnight in a dark environment. The next day, the coverslip was sealed to the edge of the slide using transparent nail polish and allowed to dry for 1 hour. This step helps to minimize movement between the coverslip and slide. Finally, the slides were imaged using a confocal microscope or stored at 4°C for future use.

RNA isolation and quantitative real-time PCR (qRT–PCR)

RNA was extracted using TRIzol (Vazyme, Nanjing, China) and reverse transcribed with the PrimeScript RT–PCR kit (Vazyme, Nanjing, China) according to the manufacturer's instructions. RT–PCR was performed using SYBR Green Master Mix (Vazyme, Nanjing, China) at thermal cycling settings: 1 initial cycle at 95 °C for 5 min followed by 40 cycles of 15 s at 95 °C and 34 s at 60 °C on a 7500 Real-time

PCR system (Applied Biosystems). GAPDH was used as an internal control. All primers used are listed in **Supplementary Table 11.**

Sequence conservation analysis

Conservation analyses of DDX3X were performed using Multiple Sequence Alignment Analysis (https://www.ebi.ac.uk/Tools/msa/clustalo/).

References:

1. Chen B, Wang M, Qiu J, Liao K, Zhang W, Lv Q, et al. Cleavage of tropomodulin-3 by asparagine endopeptidase promotes cancer malignancy by actin remodeling and SND1/RhoA signaling. *J Exp Clin Cancer Res.* 2022;41(1):209.
